# Alternative Copper-Based Single-Atom Nanozyme with Superior Multienzyme Activities and NIR-II Responsiveness to Fight against Deep Tissue Infections

**DOI:** 10.34133/research.0031

**Published:** 2023-01-13

**Authors:** Jiaxiang Bai, Yonghai Feng, Wenming Li, Zerui Cheng, Jessica M. Rosenholm, Huilin Yang, Guoqing Pan, Hongbo Zhang, Dechun Geng

**Affiliations:** ^1^ Department of Orthopedic Surgery, Orthopedic Institute, The First Affiliated Hospital, Medical College, Soochow University, Suzhou, Jiangsu 215006, P. R. China.; ^2^ Institute for Advanced Materials, School of Materials Science and Engineering, Jiangsu University, Zhenjiang, Jiangsu 212013, P. R. China.; ^3^ Pharmaceutical Sciences Laboratory, Faculty of Science and Engineering, Åbo Akademi University, Turku 20520, Finland.; ^4^ Turku Bioscience Centre, University of Turku and Åbo Akademi University, Turku 20520, Finland.

## Abstract

Nanozymes are considered to represent a new era of antibacterial agents, while their antibacterial efficiency is limited by the increasing tissue depth of infection. To address this issue, here, we report a copper and silk fibroin (Cu-SF) complex strategy to synthesize alternative copper single-atom nanozymes (SAzymes) with atomically dispersed copper sites anchored on ultrathin 2D porous N-doped carbon nanosheets (CuN*_x_*-CNS) and tunable N coordination numbers in the CuN*_x_* sites (*x* = 2 or 4). The CuN*_x_*-CNS SAzymes inherently possess triple peroxidase (POD)-, catalase (CAT)-, and oxidase (OXD)-like activities, facilitating the conversion of H_2_O_2_ and O_2_ into reactive oxygen species (ROS) through parallel POD- and OXD-like or cascaded CAT- and OXD-like reactions. Compared to CuN_2_-CNS, tailoring the N coordination number from 2 to 4 endows the SAzyme (CuN_4_-CNS) with higher multienzyme activities due to its superior electron structure and lower energy barrier. Meanwhile, CuN*_x_*-CNS display strong absorption in the second near-infrared (NIR-II) biowindow with deeper tissue penetration, offering NIR-II-responsive enhanced ROS generation and photothermal treatment in deep tissues. The in vitro and in vivo results demonstrate that the optimal CuN_4_-CNS can effectively inhibit multidrug-resistant bacteria and eliminate stubborn biofilms, thus exhibiting high therapeutic efficacy in both superficial skin wound and deep implant-related biofilm infections.

## Introduction

Compared with superficially exposed skin wounds, infections that occur at deeper sites, such as osteomyelitis [1] and implant-related and deep soft tissue infections [2–4], are exceedingly more difficult to treat with antibiotics since they are deeper and often associated with methicillin-resistant *Staphylococcus aureus* (MRSA) [5,6]. To this end, incision, drainage, and long-term high-dose antibiotic administration are usually needed. However, these courses of action are more likely to promote bacterial evolution into super strains [7]. Meanwhile, owing to inherently high reproduction and strong adherence, biofilms, known as bacterial communities, can easily form and are much harder to eliminate on the surfaces of deep sites such as implants [8,9]. The failure rate of antibiotic treatment continuously increases, and even persistent systemic infections to human hosts inevitably take place. Although antibiotic-loaded nanocarriers are able to enhance the therapeutic efficiency of antibiotics, they usually require a long time (several days) for antibiotic-administered therapeutics [10]. Therefore, it is imperative to develop antibiotic-free, in situ, and effective strategies to fight deep tissue infections.

Alternatively, nanozyme-based antibacterial therapy has emerged as a new era of powerful weapons to combat bacterial infections without causing antimicrobial resistance (AMR), since the antibacterial mechanism is based on enzyme-mimetic catalytic generation of highly toxic agents, such as reactive oxygen species (ROS) [11–13]. These agents can irreversibly and rapidly damage the cell wall, membrane, DNA and proteins of bacteria, as well as extracellular DNA and polysaccharides in biofilms [14]. Peroxidase (POD)- or oxidase (OXD)-like nanozymes have been widely developed for antibacterial applications due to the conversion of hydrogen peroxide (H_2_O_2_) or oxygen (O_2_) into hydroxyl (•OH) or superoxide anion (•O_2_^−^) radicals under biological conditions [15]. However, in deep tissues [5], the catalytic ROS generation of nanozymes certainly decreases due to limited substrate diffusion. In this case, it requires the nanozymes to possess higher activity to produce sufficient ROS up to the inhibitory levels under lower substrate concentrations when facing deep infections.

Compared to traditional noble-metal or transition-metal oxide/sulfide-based nanozymes, single-atom nanozymes (SAzymes) possess atomically dispersed metal atoms with a maximum atom utilization efficiency, remarkably enhancing the enzyme-like activities [16]. Different from noble-metal single atom-based SAzymes [17,18], transition-metal single atom and nitrogen codoped porous carbon material (MN*_x_*-C)-based SAzymes generally have similar active centers to those of natural metalloenzymes, which consist of metal and nitrogen coordination (MN*_x_*, M = Fe, Zn, Cu), resulting in extremely high or superior POD- or OXD-like activities even compared to their natural counterparts [19]. By the merits of the coordinated Cu atom serving as cofactor or active center in copper-containing metalloenzymes (e.g., laccase, urate oxidase, amine oxidase, and azurin) [20], it is interesting to find that in addition to high POD-like activity, the CuN*_x_*-C SAzyme also possesses excellent OXD-like activity, meaning it can promote ROS generation through the parallel reactions of converting H_2_O_2_ into •OH and O_2_ into •O_2_^−^ [21]. On the other hand, the Cu decoration in defective porous carbon materials can further broaden the optical absorption in the near-infrared (NIR) region and improve the photothermal conversion efficiency of the nanohybrid (48.5%) [22,23]. These results suggest that CuN*_x_*-C SAzymes are potential candidates with multiple enzyme-mimicking activities and NIR responsiveness for deep infection treatment. It is noted that the activity of CuN*_x_* sites for the CuN*_x_*-C single site catalysts significantly depends on the N-coordination number due to their different electron structures affecting their catalytic abilities [24]. However, regulating the coordination environment of CuN*_x_*-C SAzymes is vital for enzyme-mimicking reaction but still remains a challenge.

**Fig. 1.**
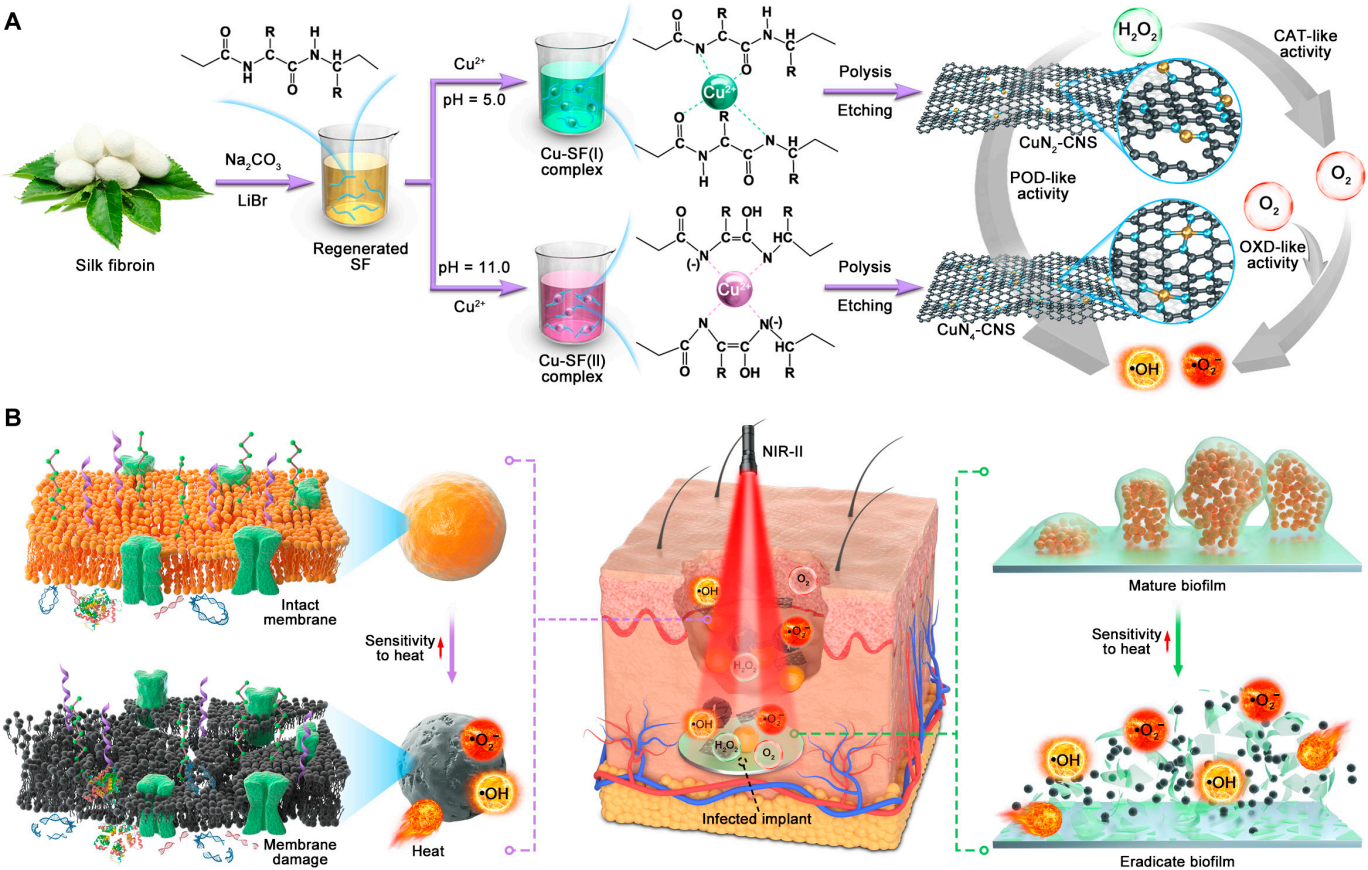
Synthesis procedure and antibacterial therapy mechanism of CuN*_x_*-CNS SAzyme. (A) Schematic illustration of CuN*_x_*-CNS synthesis based on the pyrolysis of the Cu-SF complex strategy. (B) NIR-II light-responsive and triple-enzyme-mimicking CuN*_x_*-CNS SAzyme for antibacterial therapies from superficial to deep tissue infections.

It is interesting to note that silk fibroin (SF) from *Bombyx mori* silkworm not only is a natural protein rich in nitrogen (N) and carbon (C) but also can specifically chelate Cu^2+^ to form different Cu and SF (Cu-SF) complexes, with central Cu^2+^ coordinated with different N numbers changing from 4 to 1 when the pH is reduced from 8.0 to 4.0 [25]. Inspired by this, Cu-SF complexes can be used as precursors to synthesize different CuN*_x_*-C SAzymes with tunable N coordination numbers. Thus, in this work, we developed an alternative Cu-SF complex pyrolysis strategy to fabricate alternative CuN*_x_*-C SAzymes with tunable N coordination numbers, namely, ultrathin two-dimensional (2D) N-doped porous carbon nanosheet supported Cu single atoms (CuN*_x_*-CNS, *x* = 2 or 4), as depicted in Fig. 1A. The CuN*_x_*-CNS SAzymes possess triple enzyme-like activities [POD, OXD, and catalase (CAT)] and high NIR-II-responsive photothermal activity (40.9% photothermal conversion efficiency). Experimental studies and theoretical calculations unambiguously identified that both CuN_4_ and CuN_2_ sites exhibit similar POD-like activity, but the CuN_4_-CNS SAzyme favors superior CAT- and OXD-like activity, implying more ROS generation. Therefore, the synergy between enhanced ROS generation and photothermal ablation of CuN_4_-CNS SAzyme contributes to preeminent bactericidal efficacy against *Escherichia coli*, MRSA, and biofilms. The in vivo experiments further prove that the synergistic antibacterial effect promotes wound disinfection and implant-related biofilm removal (Fig. 1B). This work highlights the engineering of efficient SAzymes for combating bacterial infections from superficial to deep tissues.

## Results and Discussion

### Synthesis and characterization

The fabrication procedure of CuN*_x_*-CNS based on a Cu-SF complex pyrolysis strategy is depicted in Fig. 1. Generally, regenerated SF aqueous solution was first prepared by dissolving SF, which was extracted from the *B. mori* cocoon by the classical protocol [26]. After the addition of Cu(II) salt, a Cu-SF complex with square planar coordination modes of Cu-2N2O [Cu-SF(I)] was formed due to the chelation between Cu^2+^ ions and SF at a pH value of 5.0, whereas a Cu-SF complex with Cu-4N mode [Cu-SF(II)] was formed at a pH of 11.0. Meanwhile, the solution color changed from light blue to dark purple (Fig. S1A) [25]. The Cu-SF complex with Cu-4N coordination was confirmed by the ultraviolet–visible (UV–Vis) absorption spectrum, which displayed a characteristic absorption peak of ca. 540 nm (Fig. S1B). SF can self-assemble into a lamella-like layer structure after the treatment of all-aqueous extraction [27]. Thus, following freeze-drying and pyrolysis, the Cu-SF complexes were in situ transformed into 2D isolated Cu atoms and CuN*_x_*-CNS. Finally, CuN*_x_*-CNS were obtained after the removal of soluble salts and copper nanoparticles (NPs) by acid etching.

**Fig. 2.**
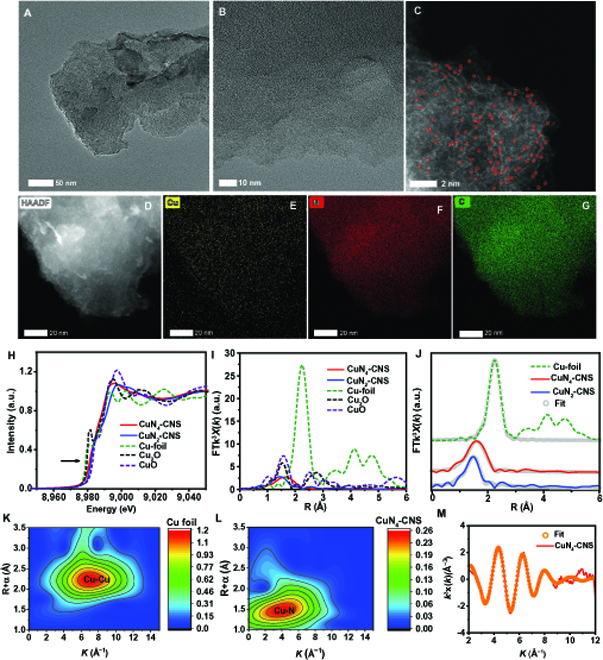
CuN*_x_*-CNS morphology characterization. (A) TEM, (B) high-resolution transmission electron microscope (HRTEM), and (C) aberration-corrected HAADF-STEM (AC-HAADF-STEM) image of CuN_4_-CNS and (D) HAADF-STEM image and (E to G) corresponding element mapping of CuN_4_-CNS: Cu (yellow), N (red), and C (green). The bright dots circled in red are single copper atoms. (H) Cu K-edge XANES spectra of CuN_4_-CNS, CuN_2_-CNS, Cu foil, Cu_2_O, and CuO. (I) Fourier transform (FT) of the Cu K-edge EXAFS spectra of CuN_4_-CNS, CuN_2_-CNS, Cu foil, Cu_2_O, and CuO. (J) EXAFS fitting result of CuN_4_-CNS, CuN_2_-CNS, and Cu foil in R space. Wavelet transform of Cu foil (K) and CuN_4_-CNS (L). (M) EXAFS fitting result of CuN_4_-CNS in *k* space.

Figure 2A shows the transition electron microcopy (TEM) of CuN_4_-CNS with a sheet-like 2D structure. Atomic force microscopy (AFM) demonstrates that the thickness of CuN_4_-CNS is approximately 1.8 nm (Fig. S2), implying a multilayer structure. Meanwhile, a number of disordered pores are clearly formed on the sheet surface because excess metal salts can provide a hypersaline environment to activate the pyrolysis process [27]. Consequently, CuN_4_-CNS displays a larger specific surface area than does the pure N-doped carbon nanosheets (N-CNS), along with a narrow pore size distribution (Fig. S3 and Table S1). As shown in the high-resolution TEM image (Fig. 2B), some irregular fringes are assigned to a few graphene layers and no obvious Cu NPs are observed. Abundant white bright dots, some circled in red, are observed on the carbon nanosheet surface from the corresponding aberration-corrected high angle annular dark field-scanning transmission electron microscope (HAADF-STEM) image (Fig. 2C), proving the presence of atomically isolated Cu single atoms on the carbon matrix. The HAADF-STEM image (Fig. 2D) and its energy-dispersive spectroscopy mappings (Fig. 2E to G) reveal that the Cu, N, and C elements are homogeneously distributed on the carbon nanosheet. In addition, the Cu content was determined to be approximately 1.5 wt% by inductively coupled plasma–optical emission spectrometry (ICP-OES) (Table S1). The x-ray diffraction (XRD) pattern (Fig. S4) of CuN_4_-CNS is similar to that of pure N-CNS, suggesting the NP-free feature of CuN_4_-CNS. X-ray photoelectron spectroscopy (XPS) demonstrates the existence of C, N, and O elements, and the high-resolution Cu 2p spectrum verifies the presence of copper species in CuN_4_-CNS (Fig. S5A). Compared to the N-CNS spectrum, the N 1s spectrum of CuN_4_-CNS can be deconvoluted into 4 obvious peaks centered at 398.2, 398.93, 400.5, and 404 eV, corresponding to pyridinic, cupric, pyrrolic, and graphitic-N species, respectively (Fig. S5B), suggesting the presence of the so-called nitrogen-coordinated Cu atom (CuN*_x_*) sites [28]. The Raman spectra (Fig. S6) show that the intensity ratios of D band to G band (*I*_D_/*I*_G_) (D, disorder; G, graphite) are calculated to be 1.03 and 0.99 for N-CNS and CuN_4_-CNS, respectively, indicating that CuN_4_-CNS is less defective than N-CNS due to the introduction of copper species, but the relatively high intensity of the D band implies that CuN_4_-CNS still has a highly defective structure [29].

X-ray absorption near-edge structure (XANES) was further used to elucidate the chemical state and coordination environment of isolated Cu atoms in CuN*_x_*-CNS. Figure 2H shows the normalized XANES curves of the Cu K-edge for CuN_2_-CNS, CuN_4_-CNS, Cu foil, Cu_2_O, and CuO. The XANES profiles of CuN*_x_*-CNS (CuN_2_-CNS and CuN_4_-CNS) are relatively smoother than those in other references, and the positions of the absorption edges are located between those of Cu_2_O and CuO, suggesting positive charges of copper valence between +1 and +2, probably caused by N doping [21]. Moreover, the Cu 2P_3/2_ XPS spectrum of CuN_4_-CNS can be deconvoluted into 2 peaks, at 933.9 and 932.5 eV, assigned to Cu(II) and Cu(I) species, respectively, which is confirmed by the corresponding Auger electron spectroscopy spectrum of Cu LMM, demonstrating that the valence state of Cu^δ+^ (1 < δ < 2) is closely correlated to the XANES analysis (Fig. S5C and D). Subsequently, extended x-ray absorption fine structure (EXAFS) was employed to determine the local coordination structure of the Cu sites of CuN*_x_*-CNS (Fig. 2I). Compared to the Cu foil reference, no scattering of the Cu–Cu coordination signal at 2.24 Å but a dominant Cu–N coordination at approximately 1.5 Å is detected in the Fourier transformed EXAFS spectrum of CuN_4_-CNS (or CuN_2_-CNS) (Fig. 2J). Additionally, the wavelet transforms of the Cu K-edge EXAFS oscillations obviously demonstrate the good atomic dispersion of Cu species in CuN_4_-CNS (Fig. 2K), with one intensity maximum of ca. 3.9 Å^−1^ assigned to the Cu–N coordination present. No intensity maximum (6.7 Å^−1^) related to Cu–Cu coordination of Cu foil (Fig. 2K) is observed. The quantitative EXAFS curve fitting (Fig. 2J and M) evidently presents a main peak related to Cu–N first shell coordination with a coordination number of ca. 3.9 ± 0.1 N atoms at a mean bond length of 2.04 ± 0.02 Å in CuN_4_-CNS (Table S2). Thus, the coordination model of Cu atoms in CuN_4_-CNS can be dominantly identified as one Cu atom coordinated by 4 N atoms (CuN_4_) confined in the carbon matrix. For CuN_2_-CNS, the morphology and structure are similar to those of CuN_4_-CNS (Figs. S2 and S7), but it has a slightly lower N content, and the CuN_2_ sites composed of one Cu atom coordinated with 2 N atoms are confirmed by quantitative EXAFS curve fitting. The above results demonstrate that the N coordination numbers of CuN*_x_*-CNS can be precisely modulated by facilely changing the coordination mode of Cu-SF complex before pyrolysis.

The UV–Vis–NIR absorption spectra (Fig. S8A) demonstrate that CuN_4_-CNS, CuN_2_-CNS, and N-CNS exhibit broad absorption ranging from the UV to the second NIR (NIR-II) biowindow, which possesses deeper tissue penetration, a higher skin tolerance threshold, and less energy dissipation compared to the first NIR (NIR-I) [30]. Significant enhancement in the optical absorption, especially in the NIR-II region, is observed for both CuN_4_-CNS and CuN_2_-CNS compared to the pure N-CNS. Therefore, upon NIR-II light (1,064 nm, 1 W cm^−2^) irradiation for 8 min, both CuN_4_-CNS and CuN_2_-CNS aqueous dispersions (200 μg ml^−1^) exhibit similar maximum temperature elevations, reaching ca. 53.3 °C from 31 °C, much higher than that of N-CNS (47.2 °C). The temperature elevation is concentration dependent (Fig. S8B), suggesting the remarkable NIR-II light-responsive photothermal activity of CuN*_x_*-CNS with a photothermal conversion efficiency of 40.9% (Fig. S8C and D). The photothermal performance of CuN_4_-CNS hardly changes during 4 heating/cooling cycles, demonstrating good photothermal stability (Fig. S8E).

### Multienzyme-mimicking activity and mechanism

**Fig. 3.**
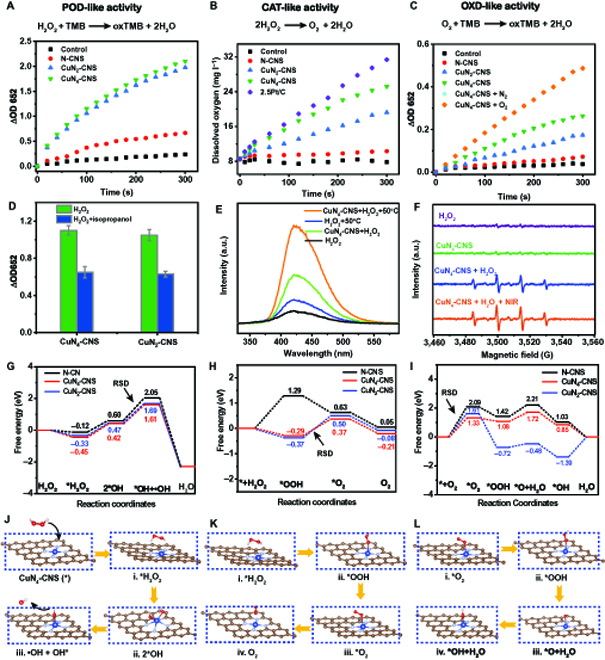
Enzyme-like catalytic performance of CuN*_x_*-CNS SAzymes. (A) POD-like activity of N-CNS, CuN_2_-CNS, and CuN_4_-CNS. (B) CAT-like activity of N-CNS, CuN_2_-CNS, and CuN_4_-CNS. (C) OXD-like activity of N-CNS, CuN_2_-CNS, and CuN_4_-CNS. (D) Isopropanol inhibition for the POD-like catalysis of CuN_2_-CNS. (E) Influences of POD-like catalysis of CuN_4_-CNS and temperature on TA oxidation. (F) ESR spectra for the detection of •OH under different conditions. (G to I) Free energy diagrams of the POD-, CAT-, and OXD-like catalysis of N-CNS, CuN_2_-CNS, and CuN_4_-CNS. (J to L) Schematic illustrations of the proposed reaction pathways of POD-, CAT-, and OXD-like catalysis.

The POD-like property of CuN*_x_*-CNS was first investigated by the typical chromogenic reaction of catalyzing the oxidation of 3,3′,5,5′-tetramethylbenzidine (TMB) to oxidized TMB (ox-TMB) in the presence of H_2_O_2_. As shown in Fig. 3A, a much higher time-dependent increase in the absorbance at 652 nm, attributable to the formation of ox-TMB, is observed for both CuN_4_-CNS and CuN_2_-CNS compared to that of N-CNS, demonstrating that both the CuN_4_ and CuN_2_ sites are highly active sites for catalyzing H_2_O_2_ decomposition into •OH. The absorbance value at 652 nm of ox-TMB obviously decreases (Fig. 3D) after adding isopropanol as an •OH scavenger into the CuN_4_-CNS (or CuN_2_-CNS)/H_2_O_2_/TMB solution, implying that •OH is produced as the active intermediate. Terephthalic acid (TA) is further used as an indicator to determine the generated •OH since it can convert the nonfluorescent TA into fluorescent 2-hydroxy terephthalic acid (HTA). Figure 3E shows that the fluorescence intensity of HTA for CuN_4_-CNS + H_2_O_2_ at room temperature is much higher than that of H_2_O_2_ alone, demonstrating the enhanced production of •OH, which can be further confirmed by the obvious electron spin resonance (ESR) signal for CuN_4_-CNS + H_2_O_2_ (Fig. 3F). Based on the steady-state kinetics of CuN_4_-CNS, the values of *K*_M_ and *V*_max_ for TMB were calculated to be 0.22 mM and 9.5 × 10^−8^ M s^−1^, while for H_2_O_2_, they were 5.6 mM and 138 × 10^−8^ M s^−1^, respectively (Fig. S9A to D). It is noted that the *K*_M_ values of CuN_4_-CNS for TMB and H_2_O_2_ are comparable to those of previously reported nanozymes, but the *V*_max_ value for catalyzing H_2_O_2_ is much higher than that of reported Cu-C-N SAzymes [31,32], even comparable to that of 2D sheet-like Fe–C–N SAzymes [33] (Table S2). As shown in Fig. 3F, the ESR signal of CuN_4_-CNS + H_2_O_2_ + NIR-II is 1.7 times higher than that without NIR-II irradiation, indicating the enhanced production rate of •OH due to the elevation of the reaction temperature. This can be proven by the significantly enhanced fluorescence intensity of HTA after TA treatment by CuN_4_-CNS + H_2_O_2_ + 50 °C (Fig. 3E). Figure S9F shows that even under weakly acidic conditions (pH 5.5), CuN_4_-CNS can still maintain high activity, which can be attributed to the participation of H^+^ ions during the reactions.

In parallel to POD, CAT can catalyze the decomposition of H_2_O_2_ to O_2_ and H_2_O. Thus, monitoring the O_2_ generated from H_2_O_2_ decomposition can reflect the CAT-like activities of CuN*_x_*-CNS. Figure 3B shows the performances of O_2_ generation for N-CNS, CuN_2_-CNS, CuN_4_-CNS, and Pt/C. Almost no O_2_ is generated without catalyst. With N-CNS as the catalyst, the dissolved O_2_ concentration only increased from 8.4 to 10.2 mg l^−1^ after reacting for 300 s. For CuN_2_-CNS, the dissolved O_2_ concentration reached 19.2 mg l^−1^ at 300 s, while it reached 25.2 mg l^−1^ for CuN_4_-CNS. Note that the dissolved O_2_ concentration of CuN_4_-CNS is close to that (31.4 mg l^−1^) of the commercial Pt/C catalyst, which is known to possess typical CAT-like activity [34], suggesting that CuN_4_ sites are more reactive than CuN_2_ sites. Furthermore, the oxygen generation velocities of the first 60 s are shown in Fig. S10, and the results indicate that the catalytic activity has a positive correlation with the CuN_4_-CNS concentration.

The OXD-like property of CuN*_x_*-CNS with the capacity to reduce O_2_ can also be detected by using TMB as a •O_2_^−^ capturer [35]. The •O_2_^−^ generation can be confirmed by using dihydroethidium (DHE) as the indicator (Fig. S11). As shown in Fig. 3C, the absorbance at 652 nm of CuN_4_-CNS is much stronger than that of CuN_2_-CNS, indicating that CuN_4_ sites have a stronger O_2_ reduction ability. In the presence of N_2_, the absorbance strength at 652 nm remarkably decreases, whereas it is evidently enhanced with continuous O_2_ supply. Based on the velocity of TMB oxidation by the OXD-like reaction, the steady-state kinetics of CuN_4_-CNS are obtained, giving *K*_M_ = 0.57 mM and *V*_max_ = 6.5 × 10^−8^ M s^−1^ for TMB (Fig. S12), comparable to those of previously reported CeO_2_ NPs, a typical OXD-like nanozyme with *K*_M_ and *V*_max_ values of 0.42 mM and 10 × 10^−8^ M s^−1^, respectively [36].

To further investigate the mechanisms of the triple enzyme-like reactions catalyzed by CuN*_x_*-CNS and clarify the effect of the N coordination number on the activities, density functional theory (DFT) calculations were used for the prediction and evaluation, which were represented in terms of the variation in free energy (Fig. 3G to I). First, 3 typical CuN_4_, CuN_2_, and NC structures in a graphene matrix were adopted as the optimized calculation models (Fig. S13). Figure S14 reveals the obvious difference in the bonding charge distributions between the central Cu coordinated with N_2_ or Cu coordinated with N_4_. Obviously, the catalytic decomposition of H_2_O_2_ into •OH over CuN*_x_*-CNS is supposed to follow a pathway of * + H_2_O_2_ → *H_2_O_2_ (i), *H_2_O_2_ → 2*OH (ii), 2*OH → •OH + *OH (iii) (Fig. 3J). The energy diagram (Fig. 3G) demonstrates that the CuN_4_ site has the highest capacity for capturing H_2_O_2_ molecules since its H_2_O_2_ adsorption energy is the lowest (−0.45 eV). Moreover, CuN_4_ also has the lowest energy barrier for the rate-determining step (RDS) (1.61 eV); thus, it is a more active site than CuN_2_ for catalyzing H_2_O_2_ decomposition into •OH. Figure 3K shows the proposed CAT-like catalytic pathway of H_2_O_2_ + * → *H_2_O_2_ (i), *H_2_O_2_ → *OOH + H^+^ + e^−^ (ii), *OOH → *O_2_ + H^+^ + e^−^ (iii), *O_2_ → O_2_ (iv). Combined with the energy diagram (Fig. 3I), it is clearly observed that the initial intermediate OOH* for O_2_ production has a more negative adsorption energy for CuN*_x_* sites (ii), suggesting that the reaction is spontaneous. Compared with the CuN_2_ site, the CuN_4_ site has a lower RDS barrier energy for forming intermediate *O_2_ (iii), and it also has easier desorption of O_2_ (iv), which can be closely associated with the higher O_2_ production from H_2_O_2_ decomposition over CuN_4_-CNS. To elucidate the enhanced OXD-like activity for CuN_4_-CNS compared to CuN_2_-CNS, the free energy diagrams for CuN_2_, CuN_4_, and NC sites are calculated on the basis of the proposed 4-electron pathway of O_2_ + * → *O_2_ (i), *O_2_ + H^+^ + e^−^ → *OOH (ii), *OOH + H^+^ + e^−^ → *O + H_2_O (iii), *O + + H^+^ + e^−^ → *OH (iv), *OH + H^+^ + e^−^ → H_2_O_2_ (v) [19], as shown in Fig. 3l. It is clearly observed that the initial O_2_ adsorption is the RDS for the whole O_2_ reduction reaction process under acidic conditions. Among them, CuN_4_ displays the lowest O_2_ adsorption energy, indicating that it has the strongest capacity to activate the O_2_ molecule. Moreover, the last step, the conversion of *OH into H_2_O (v), is more difficult for the CuN_2_ site than for the CuN_4_ site. As a result, the OXD-like activity of CuN_4_-CNS for TMB oxidation with O_2_ as an oxidant is superior to that of CuN_2_-CNS.

In general, the parallel POD- and OXD-like reactions or cascaded CAT- and OXD-like reactions contribute to enhanced ROS generation over CuN*_x_*-CNS SAzymes. However, the difference between the bonding charge distributions of CuN_2_ and CN_4_ sites causes the difference in their intrinsic enzyme-like activities, which affects the final production of ROS. Notably, despite similar POD-like activity, the CAT- and OXD-like activities of CuN_2_-CNS are much lower than those of CuN_4_-CNS. Therefore, CuN_4_-CNS can be the more desirable SAzyme for antibacterial therapy.

### Antibacterial performance

**Fig. 4.**
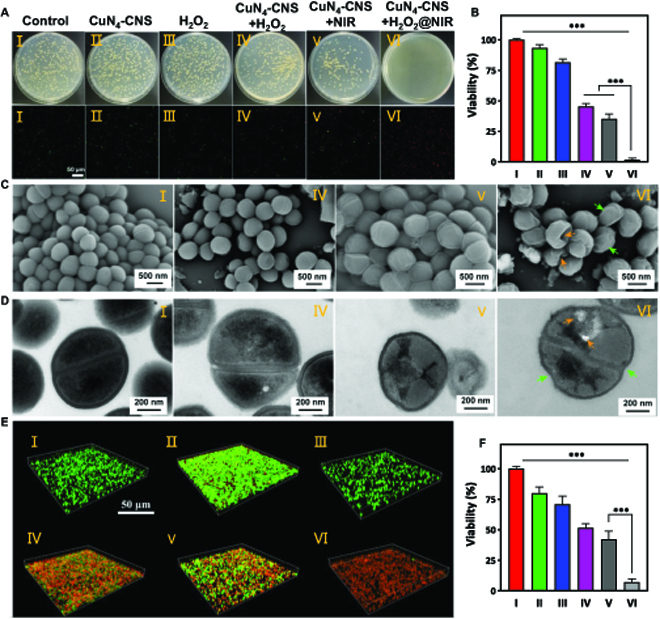
Catalytic antibacterial and antibiofilm performance of CuN_4_-CNS. (A) Photographs of bacterial colonies formed by MRSA and SYTOX/propidium iodide staining results after treatment with PBS (I), CuN_4_-CNS (II), H_2_O_2_ (III), CuN_4_-CNS + H_2_O_2_ (IV), CuN_4_-CNS + NIR (V), and CuN_4_-CNS + H_2_O_2_ + NIR (VI) for 10 min. (B) Corresponding bacterial viability of MRSA. (C and D) SEM and TEM images of MRSA after treatment with PBS (I), CuN_4_-CNS + H_2_O_2_ (IV), CuN_4_-CNS + NIR (V), or CuN_4_-CNS + H_2_O_2_ + NIR (VI). (E) Representative confocal 3D images of biofilms with green color for live bacteria and red for dead bacteria. (F) Relative amounts of corresponding biofilm biomass (*n* = 6; ***P < 0.001).

The catalytic antibacterial performances of CuN_4_-CNS against gram-negative bacteria, *E. coli*, and gram-positive bacteria, MRSA, were examined by the classical plate count method and live and dead bacterial staining method (Fig. S15A and Fig. 4A). Compared to the bacteria treated with CuN_4_-CNS (II) (200 μg ml^−1^) or H_2_O_2_ (200 μM) (III) alone, the viabilities of *E. coli* and MRSA were significantly reduced to 35.6% and 46.8% (Fig. S15B and Fig. 4B), respectively, when they were treated with CuN_4_-CNS + H_2_O_2_ (IV), which can be attributed to the catalytic ROS generation of CuN_4_-CNS inhibiting the growth of bacteria. When the laser is applied to CuN_4_-CNS, group (V), the temperature of the bacterial suspension is elevated to 48 °C upon 10 min of NIR irradiation (1,064 nm, 1 W cm^−2^), resulting in viability inhibition to 37.6% and 43.8% for *E. coli* and MRSA, respectively. It is noted that, compared to *E. coli*, the higher viability of MRSA can be attributed to the thicker cell walls or membranes of MRSA [37], endowing them with stronger tolerance to ROS attack. Interestingly, almost no *E. coli* or MRSA survived when they were treated with CuN_4_-CNS + H_2_O_2_ + NIR(VI). This can be attributed to the enhanced ROS generation of CuN_4_-CNS by an external NIR stimulus in the presence of H_2_O_2_, but more importantly in combination with local hyperthermia (even mild temperature). However, for the CuN_4_-CNS + H_2_O_2_ group (IV) or CuN_4_-CNS + NIR group (V), completely killing the bacteria requires a much higher substrate concentration (600 μM H_2_O_2_), CuN_4_-CNS loading (600 μg ml^−1^), and NIR laser power density (2 W cm^−2^) (Fig. S16). Thus, it can be seen that complete bacterial death can be achieved under the optimal conditions of 200 μg ml^−1^ CuN_4_-CNS and 200 μM H_2_O_2_ with the laser power density of 1 W cm^−2^. This enhanced antibacterial behavior of CuN_4_-CNS was further confirmed by scanning electron microscopy (SEM) and TEM. The SEM images (Fig. 4C and Fig. S15C) show that for the heathy bacterial strains treated with phosphate-buffered saline (PBS), the typically spherical or rod-shaped bacteria with smooth and intact cell walls are observed, consistent with the corresponding TEM images (Fig. 4D and Fig. S15D). However, after a single treatment of CuN_4_-CNS + H_2_O_2_ for 10 min, the bacterial surfaces of *E. coli* rapidly became rough, wrinkled, obscured, and even ruptured, and the cellular integrity could not be maintained due to cytoplasmic leakage. Despite becoming rough and obscure, the damage extent of the MRSA surface was much less than that of *E. coli*, probably due to the thicker cell membrane. For the *E. coli* treated with CuN_4_-CNS + NIR for 10 min, the bacterial surfaces became rather rough and uneven along with many holes, which resulted in serious loss of intracellular substrates, making the cell membranes crash down. For the photothermal therapy (PTT)-treated MRSA, although the outlines of the cell wall remained intact and clear, their surfaces became extremely wrinkled and looked severely dehydrated. These results demonstrate that the bactericidal mechanism of ROS attack is quite different from PTT, and the damage extent is also dependent on the type of bacteria. Interestingly, when combined (CuN_4_-CNS + H_2_O_2_ + NIR), both *E. coli* and MRSA were heavily damaged, and rather rough, uneven, wrinkled, and obscure membranes along with a number of holes were observed, therefore resulting in complete bacterial death.

### Antibiofilm performance

Considering that implant-related biofilm infections (IRIs) usually occur in deep tissue with intrinsic AMR much stronger than that of planktonic bacteria (>1,000 times) [38], it is worth investigating the catalytic antibiofilm capacity of CuN_4_-CNS. As shown in Fig. 4E and F, when the biofilm was incubated with pure CuN_4_-CNS(II), lower bacterial viability was observed compared to those treated with PBS (I) or H_2_O_2_ (III) alone (Fig. S17), which could be attributed to the OXD-like activity of CuN_4_-CNS, triggering some bacterial death in the biofilm. However, owing to the very limited generation of •O_2_^−^ through catalytic O_2_ reduction by CuN_4_-CNS, a great number of living bacteria remain inside the biofilm, which can be confirmed by the corresponding confocal 3D image. Nevertheless, with extra H_2_O_2_ addition, CuN_4_-CNS with POD-like activity can effectively catalyze the decomposition of H_2_O_2_ into abundant •OH, leading to more bacterial death inside the biofilm due to enhanced ROS generation. For the treatment with CuN_4_-CNS + H_2_O_2_ + NIR, the multienzyme activities of CuN_4_-CNS are activated and further enhanced, thus achieving significant enhancement in biofilm elimination with almost total death of bacteria. Therefore, CuN_4_-CNS can be introduced as an effective antibacterial material that not only kills planktonic bacteria but also eliminates mature biofilms.

### Biocompatibility

In addition to creating an antibacterial effect at local infection sites, the residual CuN_4_-CNS can also be exposed to healthy tissues and cells in other sites of the body. Therefore, it is also important to analyze the biocompatibility of CuN_4_-CNS to mammalian cells. Since laser and H_2_O_2_ are only applied locally, in this case, we did not study the +laser and +H_2_O_2_ groups. The results showed that no obvious viability change was observed when mammalian cells were treated with CuN_4_-CNS (<600 μg ml^−1^) (Fig. S18A), and the hemolysis measurements demonstrated a low hemolysis ratio in vitro for CuN_4_-CNS (<2%; Fig. S18A).

### Superficial skin wound healing

**Fig. 5.**
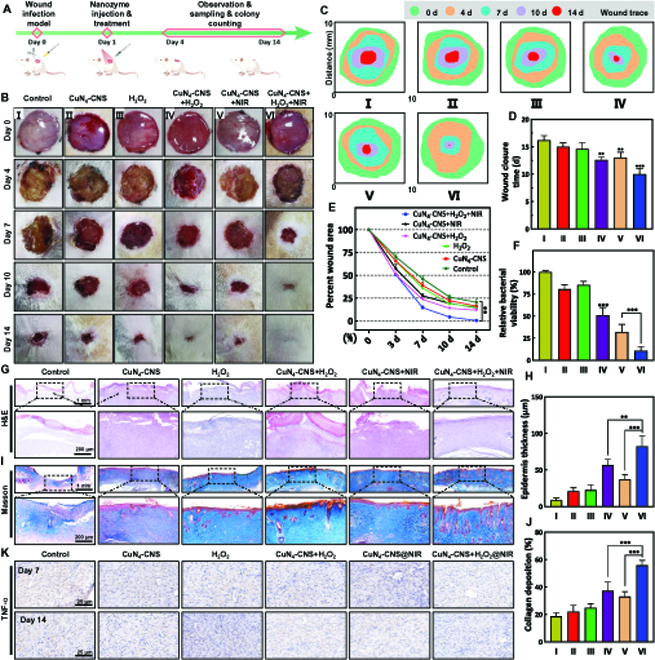
In vivo MRSA-infected wound healing effect of CuN_4_-CNS nanozyme. (A) In vivo antibacterial protocol in rats. (B) Photographs of wound tissues with different treatments on days 0, 4, 7, 10, and 14. (C) Schematic diagram of the wound healing process in vivo. (D) Wound healing rate in different groups at each time point. Wound closure time (E) and relative bacterial viability (F) in different groups. (G) H&E-stained sections of wound infection tissue in the different groups on day 7. (H) Wound epidermis thickness measured by H&E staining. (I) Masson-stained sections of wound infection tissue in each group on day 14. (J) Quantification of the collagen deposition percentage in the repaired area. (K) Representative TNF-α immunostaining images after different treatments (*n* = 6; **P < 0.01 and ***P < 0.001).

An infected rat skin wound model was subsequently constructed with MRSA and used to evaluate the therapeutic efficacy of superficial infection by CuN_4_-CNS in vivo. Figure 5A and B shows the treatment process and the healing results of superficial skin wounds treated under different conditions: PBS (I), CuN_4_-CNS (II), H_2_O_2_ (III), H_2_O_2_ + CuN_4_-CNS (IV), CuN_4_-CNS + NIR (V), or CuN_4_-CNS + H_2_O_2_ + NIR (VI) at different times (days 0, 4, 7, 10, and 14). In the gross view, a large amount of yellow pus was dispersed in the wound bed on day 4 for the control group treated with PBS, while on day 7, yellow pus decreased but could still be observed. In contrast, for all the treated groups, particularly CuN_4_-CNS + H_2_O_2_ + NIR, the wound was apparently smaller and started to dry and scab on day 7 without emerging inflammation. Especially on day 10, as illustrated in Fig. 5C to E, the wound becomes much smaller, with a relative wound area of 8%, and neither ulceration nor suppuration occurs during the treatment. On day 14, the wound was fully healed. Apart from that, the quantitative analysis of bacterial colonies from LB plates shows that the number of residual bacteria obviously decreases after treatment with CuN_4_-CNS + H_2_O_2_ + NIR (Fig. 5F) and is much lower than those of the other treated groups (II to V). It is noteworthy that the wound healing efficacy treated with CuN_4_-CNS + NIR (day 14, group V) was obviously lower than that treated with CuN_4_-CNS + H_2_O_2_, which can be attributed to the relatively low temperature, which reaches only 45 °C after NIR-II irradiation for 10 min (Fig. S19). Therefore, it is suggested that the remarkable wound healing efficacy of CuN_4_-CNS + H_2_O_2_ + NIR can predominantly originate from the enhanced ROS production of CuN_4_-CNS rather than photothermal activity when using an external NIR-II stimulus.

Hematoxylin and eosin (H&E) staining results indicated that keratinocytes migrated from the edge of the wound into the wound site in all treatment groups, especially in the CuN_4_-CNS + H_2_O_2_ + NIR group. Inflammatory cells become observably lower, granulation length significantly increases, and the epidermis gradually grows back and thickens in the normal skin sections on day 7 (Fig. 5G and H). Masson’s trichrome staining further showed that the established collagen fibers and dermal layers of wounds in the CuN_4_-CNS + H_2_O_2_ + NIR group were significantly better than those in the control group, and the collagen fibers were denser, thicker, and better arranged on day 14 (Fig. 5I and J). Figure 5K shows that the expression of tumor necrosis factor-α (TNF-α) was highest in the control group (with only bacterial injection) on day 7 and remained high after 14 days. Comparatively, the expression of TNF-α significantly decreased after treatment with CuN_4_-CNS + H_2_O_2_ + NIR. Based on this, it is inferred that the CuN_4_-CNS SAzyme can effectively alleviate the inflammatory response under infection conditions, accelerating wound healing and tissue regeneration, which may be due to the promoting effects of copper species in CuN_4_-CNS for cell migration, angiogenesis, and collagen deposition [26].

### Deep implant-associated infection treatment

**Fig. 6.**
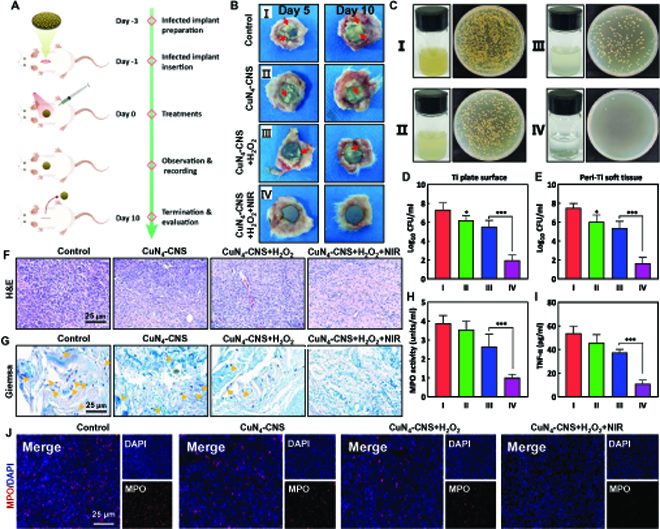
In vivo evaluation of the antibiofilm activity of CuN_4_-CNS nanozyme using the rat implant-related infection model. (A) Schematic diagram of the in vivo treatment procedure for the antibiofilm assay. (B) Bacterial burden (general observation) of implant surfaces and peri-implant soft tissues at 5 and 10 days after treatment. Red arrows indicate the white suppuration tissues in the gross specimens of implant surfaces and surrounding soft tissues. (C) Photographs of bacterial colonies in different groups (right) and the media cultured with different implants for 12 h (left). Quantitative measurements of residual biofilm on the surface of the implants (D) and residual bacteria in the peripheral tissues (E). H&E staining (F) and Giemsa staining (G) of the peri-implant soft tissues. Yellow arrows mark the residual bacteria or biofilm fragments on day 14. (H) The concentrations of TNF-α in the wound tissue were determined by ELISA on day 14. (I) Quantification of the inflammatory cell biomarkers (MPO) in peri-implant soft tissues on day 14. (J) Representative images of MPO immunofluorescence-stained sections of peri-implant soft tissues on day 14 (*n* = 6; *P < 0.05 and ***P < 0.001).

Compared with superficial skin wound infections, deep infections, including deep tissue abscesses and medical implant-associated infections (IRIs), are more troubling and concerning due to the formation of highly recalcitrant surface-associated biofilms [3,39]. Therefore, we further established a rat IRI model to validate the antibiofilm effect of CuN_4_-CNS in deep tissue in vivo. Figure 6A describes the comprehensive procedure of IRI treatment. As generally observed in the gross specimens, the implants of the untreated groups were covered by a large amount of pus and residual biofilm, especially on the 10th day after the surgery (Fig. 6B). In contrast, for the corresponding treated groups, especially with CuN_4_-CNS + H_2_O_2_ + NIR treatment, residual biofilms or pus are obviously reduced on the surfaces of implants. Figure 6C shows that the media in the CuN_4_-CNS + H_2_O_2_ + NIR group were clear and transparent, and the number of bacterial colonies was significantly lower than that in the other 3 groups. In addition, the bacterial residues on Ti plates and bacterial infiltration in the peri-implant tissues were also evaluated by estimating the number of colony-forming units (CFUs). As expected, the number of bacterial colonies in the CuN_4_-CNS + H_2_O_2_ + NIR group (IV) was considerably lower than that in the other groups, which further substantiates the prominent antibiofilm activity of CuN_4_-CNS (Fig. 6D and E). IRIs often accompany the overexpression of proinflammatory chemokines and reactions, which delay tissue repair processes. Typical acute infection signs in the control group were present, including infectious inflammatory exudation in the H&E staining (Fig. 6F) as well as myeloperoxidase (MPO) immunofluorescence staining (Fig. 6J) and biochemical analysis (Fig. 6H). Comparatively, after treatment with CuN_4_-CNS + H_2_O_2_ + NIR, an obvious decrease is observed in inflammatory infiltration. These results are consistent with the TNF-α expression by enzyme-linked immunosorbent assay (ELISA) (Fig. 6I), suggesting that CuN_4_-CNS can effectively reduce the inflammatory response. Meanwhile, numerous colonies of bacteria were visualized in the control group by Giemsa staining (Fig. 6G). In contrast, nearly no bacteria were observed in the CuN_4_-CNS + H_2_O_2_ + NIR group, but considerable bacterial colonies remained in the other 2 groups (CuN_4_-CNS + H_2_O_2_ or CuN_4_-CNS + NIR). Altogether, these results confirm that CuN_4_-CNS injection in situ with laser irradiation and H_2_O_2_ can effectively treat IRIs and have a robust inflammation resolution effect, while CuN_4_-CNS or CuN_4_-CNS + H_2_O_2_ injection can only partially reverse further deterioration of infection.

## Conclusion

In this study, a novel Cu-SF complex pyrolysis strategy was developed for the construction of CuN*_x_*-CNS SAzymes with tunable N coordination structures. The atomically dispersed CuN*_x_* sites, 2D ultrathin structure, large specific surface area, and porosity endow CuN*_x_*-CNS with excellent triple enzyme-mimetic activities, significantly promoting ROS generation through parallel POD- and OXD-like or cascaded CAT- and OXD-like catalytic pathways. The stronger O_2_ adsorption capacity and electron push effect of CuN_4_ sites contribute to the higher enzyme-mimetic activities of CuN_4_-CNS compared to those of CuN_2_-CNS. Owing to the inherently strong NIR II absorption of CuN*_x_*-CNS, using an external NIR stimulus can further promote catalytic ROS generation. As a result, the optimal CuN_4_-CNS exhibits broad-spectrum bactericidal properties and effectively inactivates the bacteria embedded in stubborn biofilms in vitro while displaying high therapeutic efficacy in treating superficial skin wound infections and deep IRIs in vivo. This work provides a new perspective for the rational design of multiple enzyme-mimicking SAzymes with great potential to combat bacterial infections from superficial to deep tissues.

## Materials and Methods

### Chemicals

*B. mori* cocoons were purchased from Huangshan Huasheng Silk Group Co. Ltd. Anhydrous sodium carbonate (Na_2_CO_3_), lithium bromide (LiBr), copper chloride dihydrate (CuCl_2_·2H_2_O), hydrochloric acid (HCl), H_2_O_2_ (30 wt%), acetic acid (HAc), and isopropanol were supplied by Sinopharm Chemical Reagent Co. Ltd., and *p*-phthalic acid (TA), 3,3',5,5'-tetramethylbenzidine (TMB), 2,2-dimethyl-3,4-dihydropyrrol-1-ium-1-olate (DMPO), and DHE were purchased from Shanghai Aladdin Bio-Chem Technology Co. Ltd.

### Synthesis of CuN*_x_*-CNS

Extracted SF was first prepared by degumming the cocoons with 1 wt% Na_2_CO_3_ solution for approximately 40 min, which was then dissolved into LiBr solution (9.3 M) for 4 h and then dialyzed for 3 days, yielding a 10 wt% regenerated silk fibroin (RSF) solution. A given amount of CuCl_2_ was added to 5 wt% RSF solution with a Cu/RSF mass ratio of 1:20. In particular, by adjusting the pH value of the mixture (5.5 or 11.0), the chelation mode between RSF and Cu^2+^ ions can be tailored. The solution was subsequently freeze-dried and pyrolyzed at 900 °C for 1 h (3 °C min^−1^) under a nitrogen atmosphere. Finally, the CuN*_x_*-CNS SAzymes were obtained after etching by HCl (1 M) at 120 °C for 12 h.

### Characterization of CuN*_x_*-CNS

The morphology of CuN_x_*-*CNS was determined by TEM (Tecnai G2 F30), AC-HAADF-STEM (FEI, USA), and AFM (MultiMode VIII SPM, Bruker). The composition and chemical states of CuN*_x_*-CNS were measured by XRD (Bruker, Germany), XAFS spectra (Shanghai Synchrotron Radiation Facility), and XPS (Kratos, England). The Cu contents were determined by using the ICPtechnique (VISTA-MPX). N_2_ adsorption–desorption isotherms were used to determine the specific surface area of CuN_x_*-*CNS on a physical adsorption apparatus (NOVA 2000e). All UV–vis and fluorescence measurements were acquired by a UV 3600 spectrophotometer and Hitachi F-4600 spectrophotometer, respectively.

### Evaluation of photothermal conversion performance of CuN*_x_*-CNS

An infrared camera (BritIR) was used to record the temperature variation of CuN*_x_*-CNS suspensions irradiated by a 1,064-nm NIR laser under different conditions. The photothermal conversion efficiency (η) of CuN*_x_*-CNS was calculated by Roper’s calculating method [40].

### Evaluation of the POD-like activity of CuN*_x_*-CNS

POD-like catalysis assays were performed in a NaAc-HAc solution mixed with H_2_O_2_ and TMB (pH 5.5). The POD-like activity of CuN*_x_*-CNS was determined by the reduction of TMB to ox-TMB, which has a characteristic absorption peak at 652 nm and exhibits blue color. The kinetic experiments were conducted by varying the concentrations of TMB and H_2_O_2_ and calculated by a typical Michaelis–Menten curve. When using TMB as substrate, the mixture solutions contained 100 μg ml^−1^ CuN*_x_*-CNS, 1 mM H_2_O_2_, and 0 to 0.2 mM TMB, while toward H_2_O_2_, the mixture solutions contained 100 μg ml^−1^ CuN*_x_*-CNS, 0.2 mM TMB, and 0 to 2.55 mM H_2_O_2_. All kinetic measurements were performed in a time-sweep fashion at 52 nm by a Shimadzu UV spectrophotometer and repeated 3 times. For pH-dependent experiments, the POD-like activities of CuN*_x_*-CNS were performed in pH of 3.0 to 8.0. Electron paramagnetic resonance (EPR) spectrometer (Bruker A300-10/12) was used to confirm the •OH generation with DMPO as spin-trapping adduct.

### Evaluation of CAT-like activity of CuN*_x_*-CNS

The CAT-mimicking property of CuN*_x_*-CNS was conducted by measuring the O_2_ produced by H_2_O_2_ dissociation using a dissolved oxygen meter (JPB-607A, INESA). Specially, 10 μl of CuN*_x_*-CNS (1 mg ml^−1^) and 50 μl of 10 M H_2_O_2_ were added into 10 ml of PBS buffer. The typical CAT-like nanozyme, namely, active carbon supported Pt NPs (2%Pt/C), was used for comparison.

### Evaluation of the OXD-like activity of CuN*_x_*-CNS

OXD-like activities were also determined by colorimetric assays with TMB as substrate. Typically, CuN*_x_*-CNS (50 μg ml^−1^) were added to TMB (800 μM) air-saturated buffer (2.0 ml, pH 5.5). The resulting ox-TMB with absorption peak at 652 nm was monitored. DHE can be used as a fluorescent indicator toward •O_2_^−^. In the test, a mixture composed of CuN*_x_*-CNS (1 mg ml^−1^) and DHE (5 μM) was reacted in air-saturated buffer (pH 5.5) for 10 min. Then, the fluorescence spectrum of DHE was recorded.

### In vitro and in vivo experiment

The experimental details were demonstrated in the Supplementary Materials.

### Statistical analysis

All data were raw data and expressed as the mean ± standard deviation (SD) with at least 3 replicates for every experimental sample. One-way analysis of variance with Tukey’s test was used for statistical analysis. The statistical analysis was conducted using Origin 9.0 software. Statistical differences were defined as *P < 0.05, **P < 0.01, and ***P < 0.001.

## Data Availability

Data supporting the findings of this study can be obtained from the corresponding author upon request.

## Supplementary Materials

Supplementary Materials Supplementary Text Fig. S1. Colors and UV–vis absorption spectrum of Cu-SF complexes. Fig. S2. AFM images of CuN*_x_*-CNS. Fig. S3. N2 absorption and desorption profile of CuN_4_-CNS and pore size distribution. Fig. S4. XRD pattern of CuN_4_-CNS and N-CNS. Fig. S5. XPS spectra of CuN_4_-CNS. Fig. S6. Raman spectra of CuN_4_-CNS and NCNS. Fig. S7. Morphology and structural characterizations of CuN_2_-CNS. Fig. S8. Photothermal conversion performance of CuN*_x_*-CNS. Fig. S9. Catalytic kinetics of CuN_4_-CNS. Fig. S10. O_2_ generation rates at different CuN_4_-CNS concentrations. Fig. S11. Fluorescence intensity of DHE treated with or without CuN_4_-CNS and CuN_2_-CNS in air-saturated solution. Fig. S12. Catalytic kinetics of CuN_4_-CNS with O_2_ as substrates. Fig. S13. Optimized structures of N-CNS, CuN_2_-CNS, and CuN_4_-CNS based on XAFS analysis. Fig. S14. Bonding charge distributions of the NC, CuN_2_, and CuN_4_ sites. Fig. S15. Catalytic antibacterial performance of CuN_4_-CNS for *E. coli*. Fig. S16. MRSA viabilities. Fig. S17. Representative culture photographs of bacterial colonies from biofilms treated by different conditions. Fig. S18. In vitro biocompatibility of CuN_4_-CNS. Fig. S19. Thermal images of rats. Table S1. Specific surface areas (*S*_BET_) calculated by the BET method based on N_2_ adsorption and desorption measurements and elemental compositions of CuN*_x_*-CNS estimated from XPS and ICP measurements. Table S2. EXAFS fitting parameters at the Cu K-edge for various samples (*Ѕ*_0_^2^ = 0.84). Table S3. Comparisons of the activities of different SAzymes.
