# Supplementary material for: Alternative Copper-Based Single-Atom Nanozyme with Superior Multienzyme Activities and NIR-II Responsiveness to Fight against Deep Tissue Infections
Source: Research (Wash D C). 2023 Jan 13;6:0031. doi: 10.34133/research.0031 (PMC10076008; doi:10.34133/research.0031)
Supplement: Supplementary Materials — Supplementary Text Fig. S1. Colors and UV–vis absorption spectrum of Cu-SF complexes. Fig. S2. AFM images of CuNx-CNS. Fig. S3. N2 absorption and desorption profile of CuN4-CNS and pore size distribution. Fig. S4. XRD pattern of CuN4-CNS and N-CNS. Fig. S5. XPS spectra of CuN4-CNS. Fig. S6. Raman spectra of CuN4-CNS and NCNS. Fig. S7. Morphology and structural characterizations of CuN2-CNS. Fig. S8. Photothermal conversion performance of CuNx-CNS. Fig. S9. Catalytic kinetics of CuN4-CNS. Fig. S10. O2 generation rates at different CuN4-CNS concentrations. Fig. S11. Fluorescence intensity of DHE treated with or without CuN4-CNS and CuN2-CNS in air-saturated solution. Fig. S12. Catalytic kinetics of CuN4-CNS with O2 as substrates. Fig. S13. Optimized structures of N-CNS, CuN2-CNS, and CuN4-CNS based on XAFS analysis. Fig. S14. Bonding charge distributions of the NC, CuN2, and CuN4 sites. Fig. S15. Catalytic antibacterial performance of CuN4-CNS for E. coli. Fig. S16. MRSA viabilities. Fig. S17. Representative culture photographs of bacterial colonies from biofilms treated by different conditions. Fig. S18. In vitro biocompatibility of CuN4-CNS. Fig. S19. Thermal images of rats. Table S1. Specific surface areas (SBET) calculated by the BET method based on N2 adsorption and desorption measurements and elemental compositions of CuNx-CNS estimated from XPS and ICP measurements. Table S2. EXAFS fitting parameters at the Cu K-edge for various samples (Ѕ02 = 0.84). Table S3. Comparisons of the activities of different SAzymes. [file research.0031.f1.docx]

**Alternative Copper-based Single-atom Nanozyme with Superior Multienzyme activities and NIR-II Responsiveness to Fight Against Deep Tissue Infections**

Jiaxiang Bai^1^, Yonghai Feng^2,^*, Wenming Li^1^, Zerui Cheng^2^, Jessica M. Rosenholmc^3^, Huilin Yang^1^, Guoqing Pan^2,^*, Hongbo Zhang^3,4,^* and Dechun Geng^1,^*

* **Correspondence should be addressed to** Dechun Geng: [szgengdc@suda.edu.cn](mailto:szgengdc@suda.edu.cn); Hongbo Zhang: [hongbo.zhang@abo.fi](mailto:hongbo.zhang@abo.fi); Guoqing Pan: [panguoqing@ujs.edu.cn](mailto:panguoqing@ujs.edu.cn) and Yonghai Feng: [fengyonghai@ujs.edu.cn](mailto:fengyonghai@ujs.edu.cn)

^1^ Department of Orthopedic Surgery, Orthopedic Institute, The First Affiliated Hospital, Medical College, Soochow University, Suzhou, Jiangsu 215006, P. R. China.

^2^ Institute for Advanced Materials, School of Materials Science and Engineering, Jiangsu University, Zhenjiang, Jiangsu 212013, P. R. China.

^3^ Pharmaceutical Sciences Laboratory, Faculty of Science and Engineering, Åbo Akademi University, Turku 20520, Finland

^4^ Turku Bioscience Centre, University of Turku and Åbo Akademi University, Turku 20520, Finland

**This PDF file includes:**

Supplementary Text

Figures. S1 to S11

Tables S1 to S3

Supplementary Text

Figure S1. Colors and UV‒vis absorption spectrum of Cu-SF complexes.

Figure S2. AFM images of CuNx-CNS.

Figure S3. N2 absorption and desorption profile of CuN_4_-CNS and pore size distribution.

Figure S4. XRD pattern of the CuN_4_-CNS and N-CNS.

Figure S5. XPS spectra of CuN_4_-CNS.

Figure S6. Raman spectra of CuN_4_-CNS and NCNS.

Figure S7. Morphology and structural characterizations of the CuN_2_-CNS.

Figure S8. Photothermal conversion performance of the CuN_x_-CNS.

Figure S9. Catalytic kinetics of CuN_4_-CNS**.**

Figure S10. O2 generation rates at different CuN_4_-CNS concentrations.

Figure S11. Fluorescence intensity of DHE treated with or without CuN_4_-CNS and CuN_2_-CNS in air-saturated solution.

Figure S12. Catalytic kinetics of CuN_4_-CNS with O2 as substrates.

Figure S13. Optimized structures of N-CNS, CuN_2_-CNS, CuN_4_-CNS based on XAFS analysis.

Figure S14. Bonding charge distributions of the NC, CuN_2_ and CuN_4_ sites.

Figure S15. Catalytic antibacterial performance of the CuN_4_-CNS for *E. coli*.

Figure S16. MRSA viabilities.

Figure S17. Representative culture photographs of bacterial colonies from biofilms treated by different conditions.

Figure S18. In vitro biocompatibility of CuN_4_-CNS.

Figure S19. Thermal images of rats.

Table S1. Specific surface areas (S_BET_) calculated by the BET method based on N_2_ adsorption and desorption measurements and elemental compositions of the CuN_x_-CNS estimated from XPS and ICP measurements.

Table S2. EXAFS fitting parameters at the Cu K-edge for various samples (*Ѕ*_0_^2^= 0.84)

Table S3. Comparisons of the activities of different SAzymes.

**Supplementary Materials and Methods**

***Theoretical calculation description.*** The DFT calculations were conducted by the Vienna Ab initio Simulation Package (VASP). The exchange-correlation effects were described by the Perdew-Burke-Ernzerhof (PBE) functional within the generalized gradient approximation (GGA) method. The core-valence interactions were accounted for by the projected augmented wave (PAW) method. The energy cutoff for plane wave expansions was set to 420 eV, and the 2×2×1 Monkhorst-Pack grid k-points were selected to sample the Brillouin zone integration. The vacuum space is adopted 15 Å above the surfaces to avoid periodic interactions. The structural optimization was completed for energy and force convergence set at 1.0×10^-4^ eV and 0.02 eV Å^-1^, respectively. The Gibbs free energy change (ΔG) of each step is calculated using the following formula:

∆G = ∆E + ∆ZPE - T∆S

where ΔE is the electronic energy difference directly obtained from DFT calculations, ΔZPE is the zero point energy difference, T is the room temperature (298.15 K) and ΔS is the entropy change. ZPE can be obtained after frequency calculation by:

ZPE =$\frac{1}{2} \sum hvi$

The TS values of adsorbed species are calculated according to the vibrational frequencies:

$$TS = k_{B}T [ \sum_{k} ln(\frac{1}{1-e^{-hv/k_{B}T}})+ \sum_{k} \frac{hv}{k_{B}T} \frac{1}{{(e}^{hv/k_{B}T}-1)}+1 ]$$

***Catalytic antibacterial test.*** *MRSA* (ATCC43300) supplied from Affiliated Hospital of Jiangsu University was chosen as the model of Gram-positive bacteria, while the *E. coli* (ATCC25922) supplied from the Institute of Life Science of Jiangsu University were selected as the model of Gram-negative bacteria. The bacteria were incubated separately with six different groups: (I) PBS, (II) H_2_O_2_, (III) CuN_4_-CNS, (IV) CuN_4_-CNS+H_2_O_2_, (V) CuN_4_-CNS+NIR, and (VI) CuN_4_-CNS+H_2_O_2_+NIR. Concentrations: CuN_4_-CNS 200 μg mL^−1^, H_2_O_2_ 100 μM. An1064 nm NIR laser was used as the light source. The bacterial concentration was measured by using the standard spread plate method.

***Live/dead staining test.*** The model bacteria were grown overnight and washed with equal saline twice to ensure that the OD_600_ was 1.3. Then, the suspension of MRSA was incubated with CuN_4_-CNS in the absence or presence of H_2_O_2_ or NIR irradiation. Equal propidium iodide and Syto9 (L-7012, LIVE/DEAD BacLight Bacterial Viability Kit, Molecular Probes, OR, USA) were added to saline. The supernatant of bacteria and CuN_4_-CNS was discarded by centrifugation at 4000 rpm for 1 min, and dye molecules that permeated the bacterial membrane fluorescent dyes were selected for use with LIVE/DEAD@BacLight™ Bacterial Viability Kits (L7012), purchased from Molecular Probes, OR, USA. Here, 0.1 μL of SYTO9 (3.34 mM, 300 μL) and propidium iodide (PI) (20 mM, 300 μL) was dissolved in 1 mL of 0.8% saline solution before use. Then, 100 μL bacterial suspension (OD_600_ = 1.2) was mixed with 300 μL CuN_4_-CNS suspension (200 μg mL−1) and 10 μL H_2_O_2_ solution (4 mM) in a 4 mL centrifuge tube, which was irradiated with or without a 1064 nm NIR laser at 1 W cm−2. This was followed by centrifugation at 4000 rpm to remove superfluous CuN_4_-CNS from the bacterial solution. Finally, 400 μL of SYTO9 and PI miscible liquids were added to the solution treated above and incubated for 15 min (in the dark). Next, we concentrated the solution to 100 μL, dripped 10 μL onto the slide and covered it with the coverslip using dust-free paper to remove the residue at the edges before observation using a fluorescence microscope.

***Morphology of bacteria.*** For the SEM assay, after treatment with CuN_4_-CNS+H_2_O_2_ (IV), CuN_4_-CNS+NIR (V), CuN_4_-CNS+H_2_O_2_+NIR (VI), *MRSA* and *E. coli* were harvested by centrifugation and washed 3 times using PBS. After fixation with 4% paraformaldehyde containing phosphate-buffered saline (PBS) solution for 12 h at 4 °C. Then, the bacteria were dehydrated by sequential treatments with 10, 30, 50, 70, 90, and 100% ethanol for 30 min. The bacteria were dried to obtain SEM images. For the TEM assay, after the process of fixation, the bacteria were rinsed with PBS three times, treated with osmium tetroxide, and dehydrated successively in an alcohol series. Then, these samples were embedded, cut into 60 nm-thick slices, and placed on bare 200 mesh copper grids. Afterward, these sections were stained using 2% uranyl acetate and dried in a desiccator. Finally, the grids were examined using a JEOL 1200Ex TEM at 80 kV.

***Biofilm culture.*** Stationary growth phase MRSA (10 μL) was mixed with TSB medium (990 μL, 3%, containing 1% glucose) in 24-well plates at 37 °C for 48 h. The medium was refreshed every 24 h. Finally, unbound bacteria and TSB medium were removed by washing with PBS buffer, and the biofilm attached to the 24-well plates was harvested.

***Catalytic antibiofilm test.*** The as-prepared biofilm was incubated with CuN_4_-CNS or CuN_4_-CNS+H_2_O_2_ for 1 h. For the light irradiation groups, 1064 nm laser irradiation was performed on each plate. To determine the viability of bacteria in the residual biofilms, the biofilms were stained with BacLight live/dead dye in the dark for 15 min. After incubation, the redundant dye was washed with saline three times. Finally, the biofilms were observed under a CLSM (Leica TCS SP5). Additionally, the biofilms were sonicated and dispersed in 1 mL PBS buffer. Finally, the residual bacteria in the biofilm could be counted on LB agar plates.

***In vitro biocompatibility of the CuN_4_-CNS.*** A CCK-8 assay was used to evaluate the cytotoxicity of CuN_4_-CNS. Briefly, 1 × 10^4^ human umbilical vein endothelial cells (HUVECs) were cocultured with different concentrations (0-500 μg/ml) of CuN_4_-CNS samples in a 24-well plate. After 24 h, the CCK-8 reagents were added for 2 h. Optical density was measured at 450 nm and normalized to the PBS control. All of the tests were repeated three times.

A hemolysis activity assay was performed to evaluate the blood compatibility of the CuN_4_-CNS. Specifically, 500 μl of rat blood was collected and centrifuged at 4 °C and 3500 rpm for 5 min. The wash step was repeated three times until the supernatant was clear. Then, different concentrations (0-500 μg/ml) of CuN_4_-CNS samples were mixed with 1 mL erythrocytes in a tube at room temperature for 4 h. A negative control (PBS) and a positive control (pure water) were also included in the experiment to calculate the degree of hemolysis. All of the samples were centrifuged at 3500 rpm for 5 min, and the upper solution was taken to detect the absorbance at 545 nm by a microplate reader.

***In vivo rat skin superficial infection and healing process.*** All animal experiments were reviewed and approved by the Animal Investigation Ethics Committee of Soochow University (202109A0512). To investigate the efficacy of the CuN_4_-CNS in shallow tissue bacterial infections in vivo, 36 Sprague Dawley (SD) rats (6-8 weeks) were employed in this experiment (n=6). A round full-thickness cutaneous wound with a diameter of approximately 10 mm was created on the back of each rat. A suspension (200 µL) containing MRSA (10^6^ CFU/mL) was placed on the wounds. After being infected, the rats were divided into 6 groups with different treatments. The wounds were observed and photographed, and medical gauze was replaced at each expected time point. The dosage of H_2_O_2_ used in this study was 50 µL (10 mM) per site. The dosage of CuN_4_-CNS nanozymes was 50 µL per site (100 µg/mL). At days 7 and 14, three rats were sacrificed, their fresh wound tissues were collected for histological and immunofluorescence analysis, and the numbers of bacteria in them were determined by plate count methods. Relative bacteria number = CFU (test group)/CFU (PBS group) × 100%.

***Biofilm of implant preparation in vivo:*** Briefly, 500 μL of prepared MRSA solution (1×10^6^ CFUs/ml) was coincubated with sterile titanium plates under static conditions for 48 hours (in 24-well plates at 37 °C). Half of the culture medium was carefully replaced with fresh TSB after 24 h. After 48 h, the titanium plates, including biofilms, were harvested and rinsed twice with PBS. Then, they were stored at 4 °C for in vivo deep wound infection (infection around the implant) experiments.

***In vivo rat implant-associated infection and healing process.*** To investigate the efficacy of CuN_4_-CNS in deep wound infections in vivo, we used a rat model of foreign-body infection, which closely mimics human implant-associated infections. Briefly, twenty-four SD rats were employed and divided into 4 groups with different treatments (n=6): PBS (I), CuN_4_-CNS (II), CuN_4_-CNS+H_2_O_2_ (III), and CuN_4_-CNS+H_2_O_2_+NIR. (IV). Prior to the operation, the rat was anesthetized by an intraperitoneal injection of pentobarbital, the skin of the back was shaved, and a 1.5 cm long incision was made on the back. Then, the preprepared biofilm sample (15 mm × 15 mm × 1 mm) was inserted subcutaneously, and the skin incision was gently closed. The nanozymes (100 μg/ml, 50 μl) were administered in situ via cavity injection. At days 5 and 10, three rats per group were sacrificed, and the subcutaneous implants with the peri-implant soft tissues were aseptically harvested.

***Histological evaluation of the tissues.*** Harvested tissues were fixed with paraformaldehyde (4%) after washing with 0.9% NaCl solution and dehydrated. Subsequently, histological sections were obtained after embedding the skin tissues in paraﬃn. H&E staining was utilized to evaluate the inﬁltrated inﬂammatory status and observe the inﬁltration of inﬂammatory cells in skin tissues. Giemsa staining was used to distinguish bacteria from cells. In addition, histological sections were deparaffinized, washed three times in PBS for 5 min, and blocked with serum (5%) for 30 min. Then, the sections were incubated with rabbit anti-TNF-α (1:1000 dilution, ab307164, abcam) or anti-MPO (1:10000 dilution, ab300650, abcam) antibody at 4 °C overnight. For immunohistochemical staining, Secondary antibodies (VECTOR, USA) were selected according to the host of the primary antibodies. After diaminobenzidine (DAB) staining and mounting with quick-hardening mounting medium, the specimens were observed under an optical microscope to assess the expression of different markers. And for immunohistofluorescence staining, the sections were incubated with fluorescent secondary antibody (1:1000 dilution, ab150075, abcam) for 2 h at room temperature. Quantitative analysis was performed using ImageJ software (version 1.51a, NIH).

**Supplementary Figures**

^
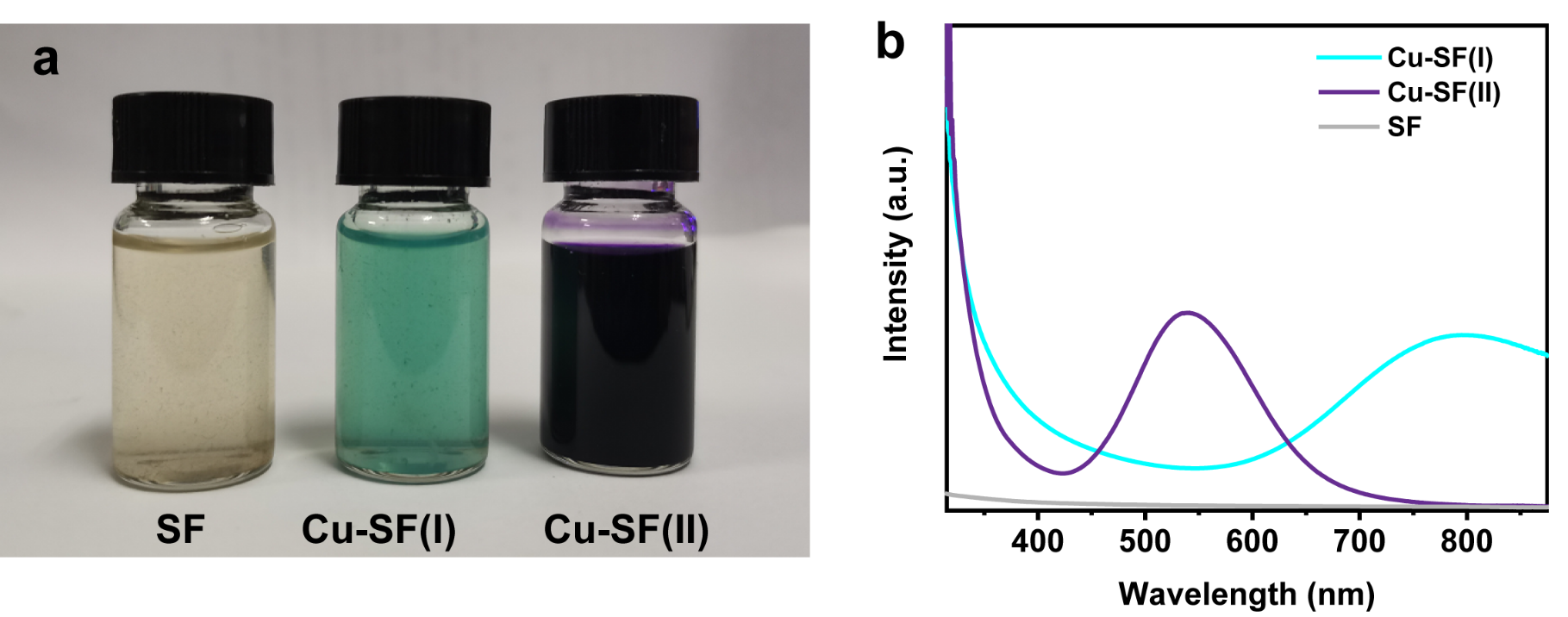
^

**Figure S1. Colors and UV‒vis absorption spectrum of Cu-SF complexes.** (a) Photograph of SF aqueous solution, and Cu-SF(I), and Cu-SF(II) complexes aqueous solutions prepared at pH = 5.0, and 11, respectively. (b) Corresponding UV‒vis absorption spectra.


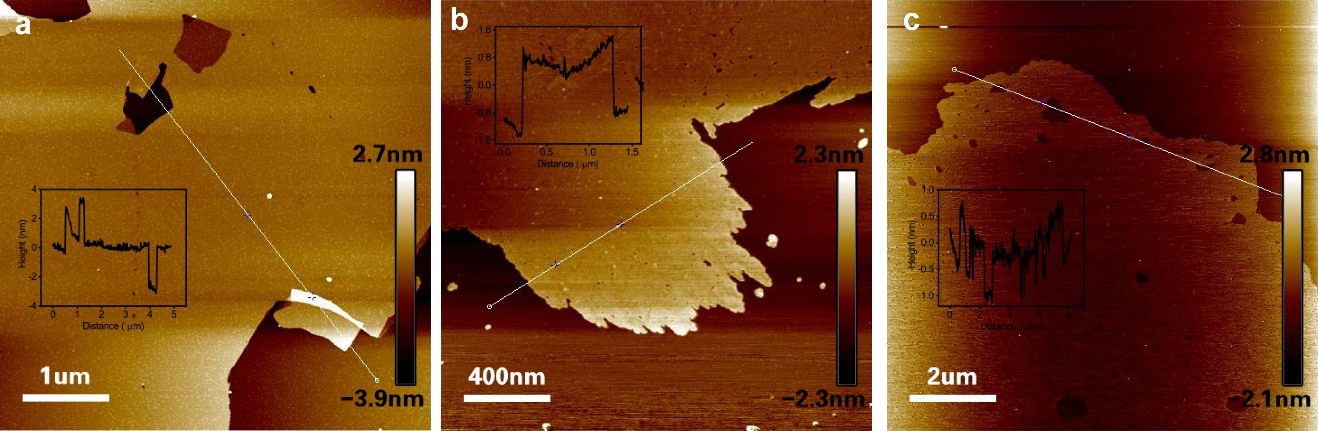


**Figure S2.** **AFM images of CuN_x_-CNS.** (a) N-CNS, (b) CuN_2_-CNS, and (c) CuN_4_-NCNS.


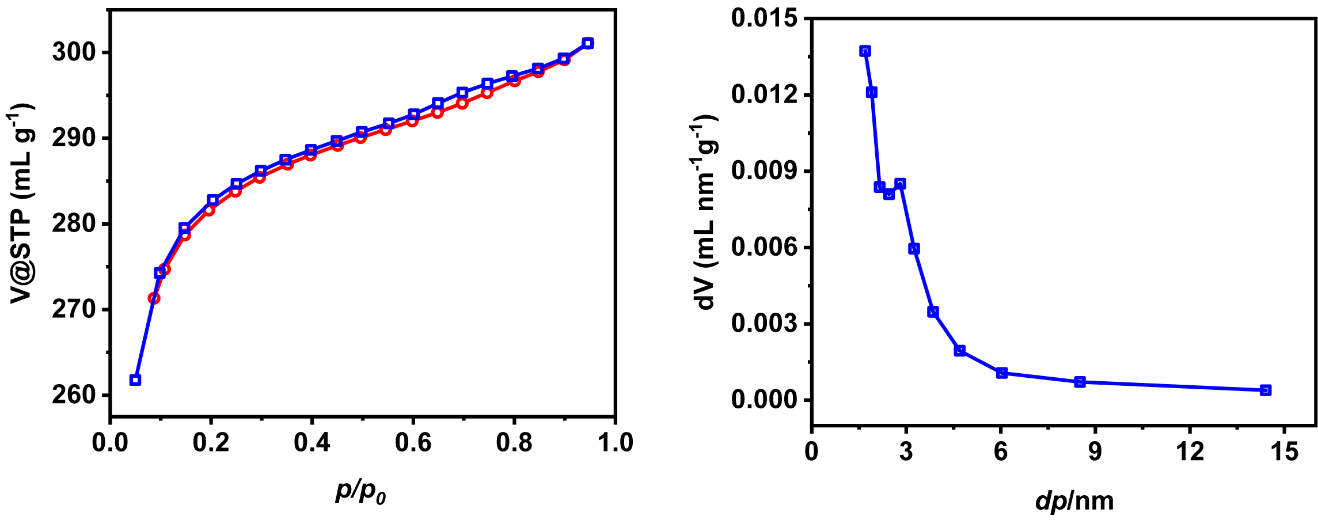


**Figure S3.** N_2_ absorption and desorption profile of CuN_4_-CNS (left) and pore size distribution (right).


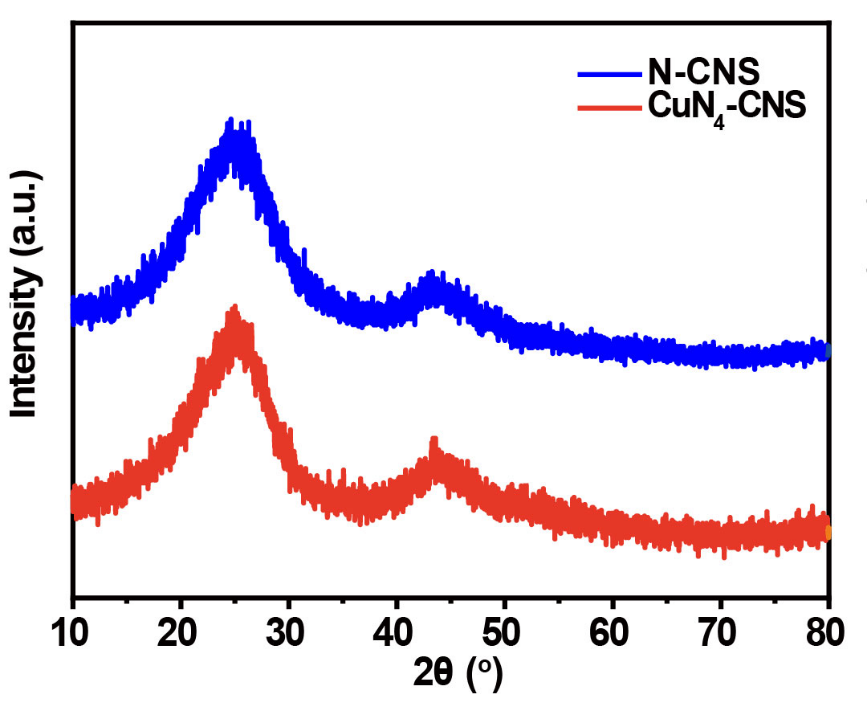


**Figure S4. XRD pattern of the CuN_4_-CNS and N-CNS.**


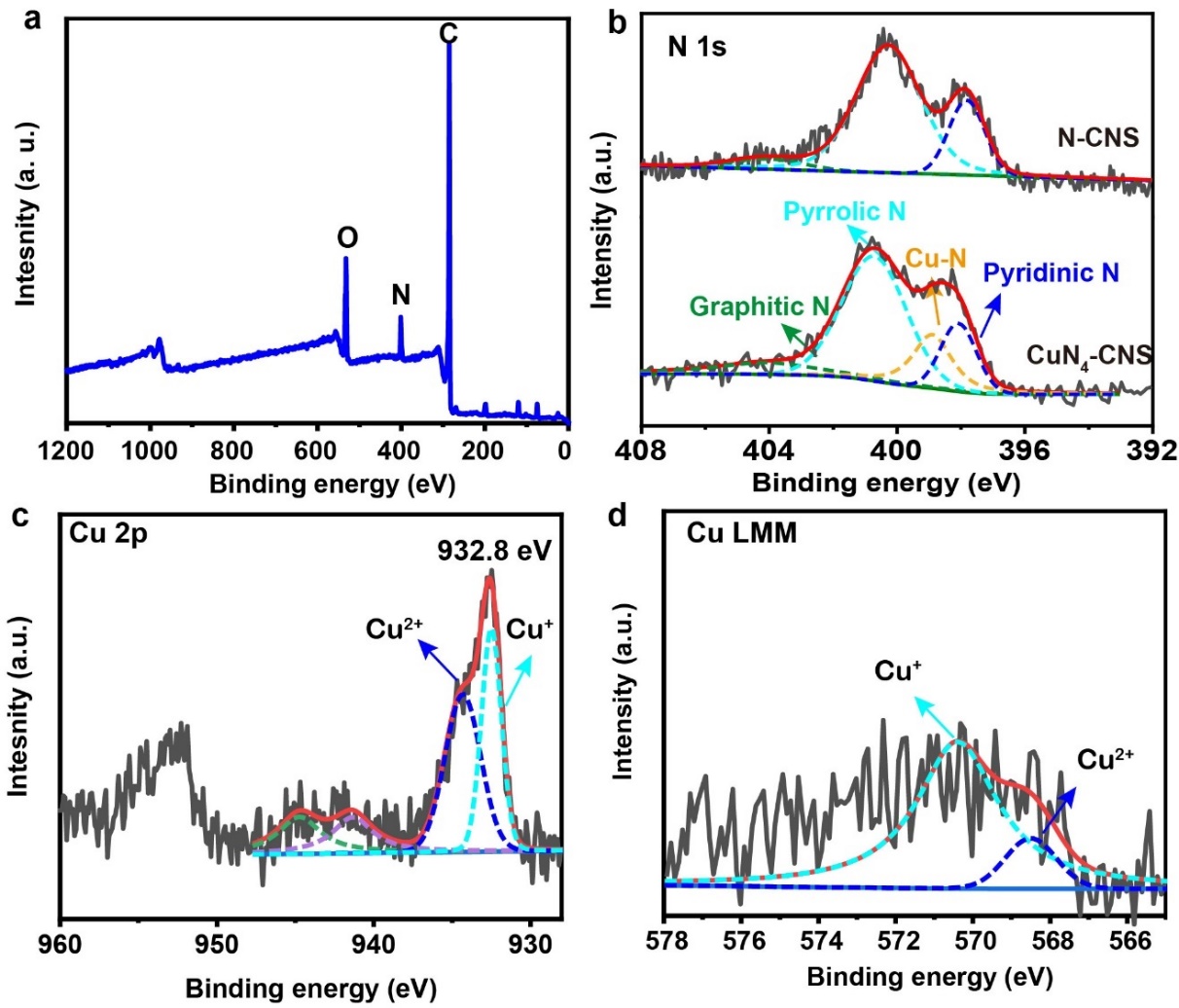


**Figure S5. XPS spectra of CuN_4_-CNS.** (a) XPS survey spectrum. (b) N 1 s XPS spectrum. (c) Cu 2P spectrum with deconvoluted Cu^2+^ and Cu^+^ peaks. (d) AES spectrum of Cu LMM with deconvoluted Cu^2+^ and Cu^+^ peaks.


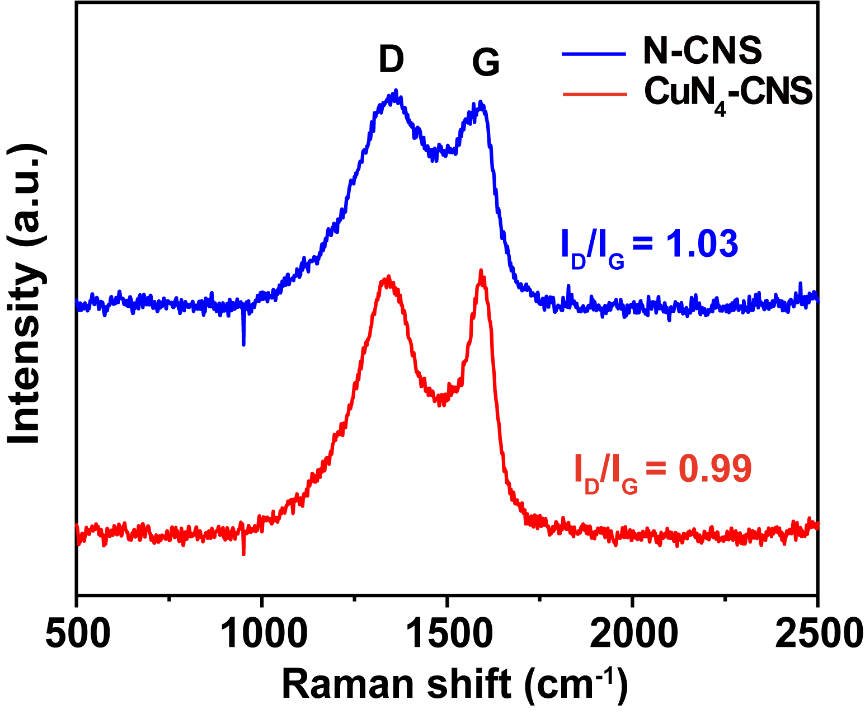


**Figure S6.** Raman spectra of CuN_4_-CNS and NCNS.


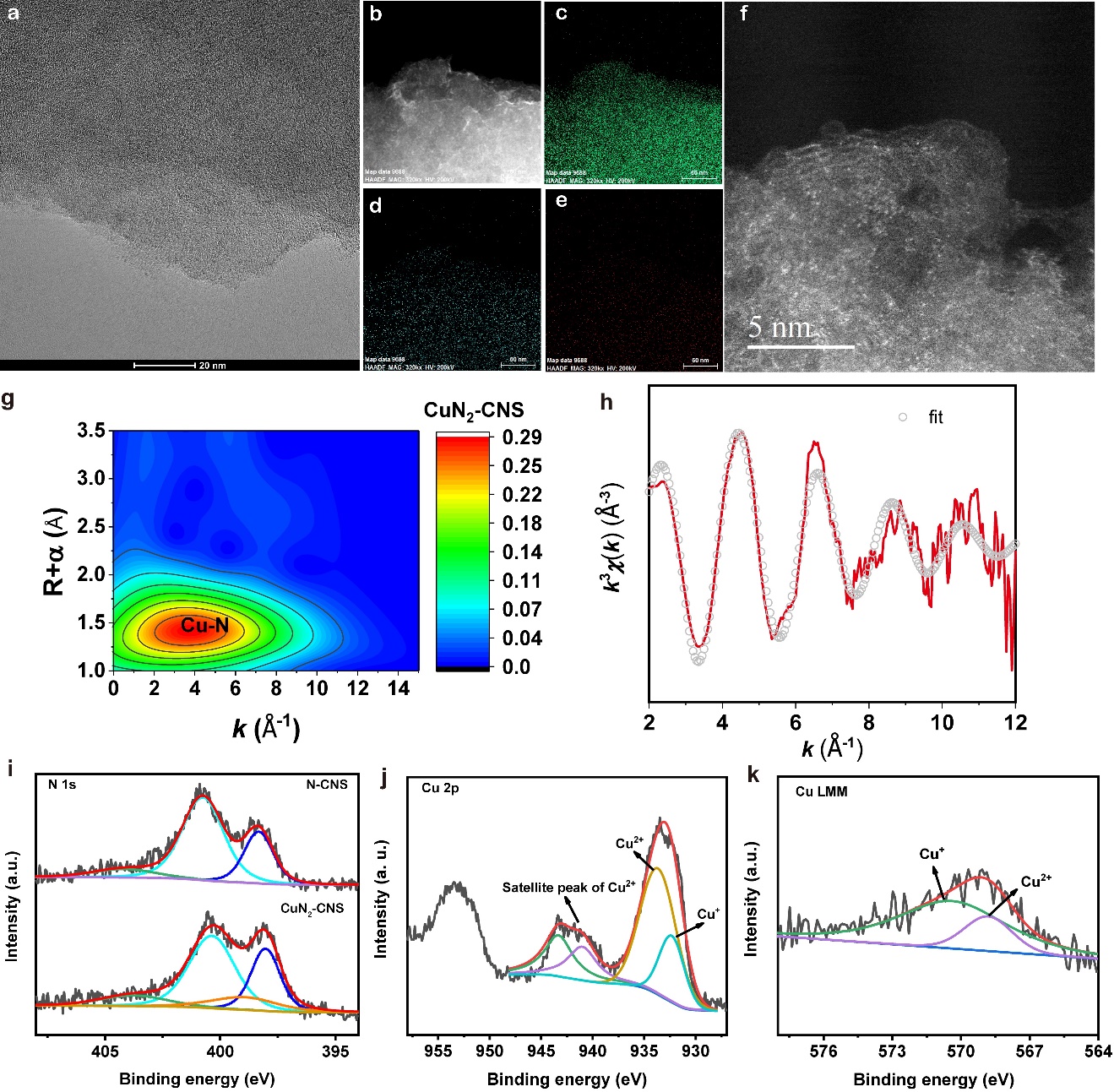


**Figure S7. Morphology and structural characterizations of the CuN_2_-CNS.** (a) HRTEM image of the CuN_2_-CNS. (b-e) HAADF-STEM image and corresponding EDS mapping of CuN_2_-CNS: C (green), N (light blue), Cu (red). (f) AC-HAADF-STEM image of the CuN_2_-CNS. (g) Cu K-edge XANES spectra of the CuN_2_-CNS. (h) EXAFS fitting result of the CuN_2_-CNS in R space. (i) Wavelet transform of the CuN_2_-CNS. N 1 s (j) and Cu 2p (k) XPS spectra of the CuN_2_-CNS. (l) AES spectrum of the Cu LMM of the CuN_2_-CNS.


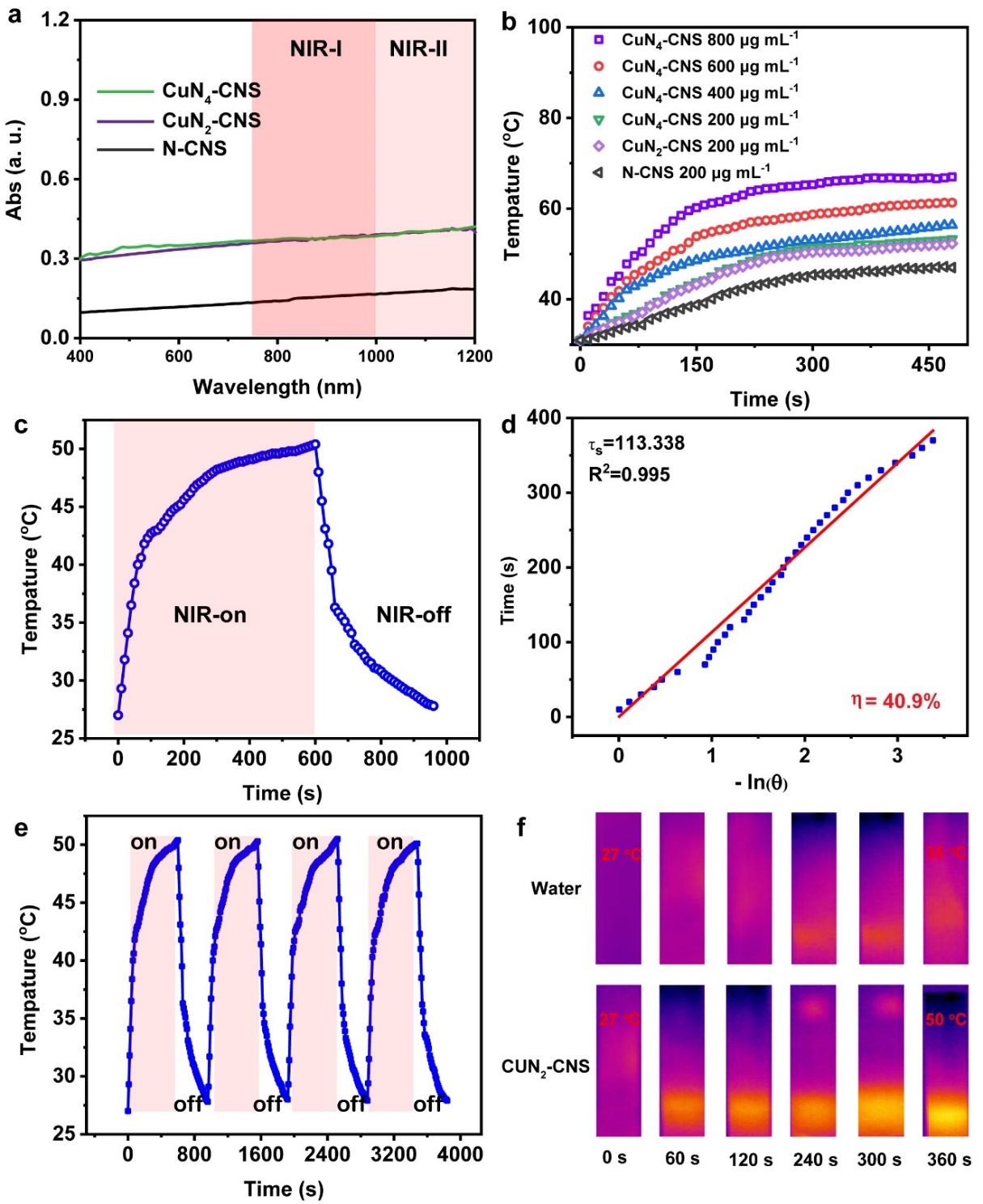


**Figure S8.** **Photothermal conversion performance of the CuN_x_-CNS.** (a) UV‒Vis-NIR absorption spectra of the CuN_x_-CNS at different concentrations. (b) Temperature elevation of the CuN_x_-CNS under NIR laser (1064 nm, 1 W cm^-2^) irradiation at different concentrations. (c) Temperature elevation and decline curves of the CuN_4_-CNS with NIR laser irradiation and shutting off. (d) The time constant for heat transfer from the system is determined to be τ_s_ = 113.3 s by applying the linear time data from the cooling period (after 600 s) versus the negative natural logarithm of the driving force temperature. (e) Heating curves of the CuN_4_-CNS with 4 laser on/off cycles. (f) Thermal imaging photographs of the CuN_4_-CNS under laser irradiation.


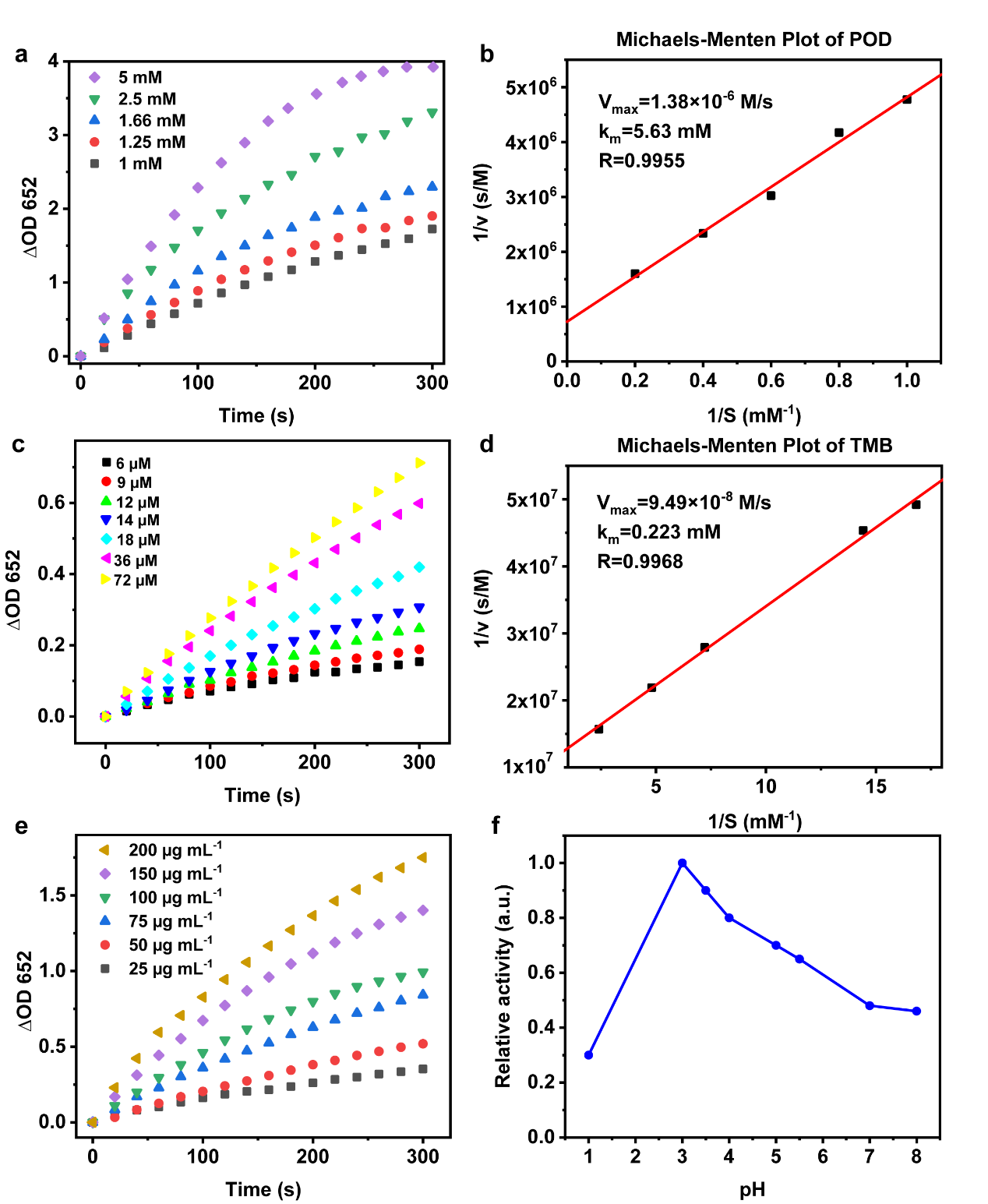


**Figure S9. Catalytic kinetics of CuN4-CNS.** (a) Velocity of the reaction measured by CuN_4_-CNS toward various concentrations of H_2_O_2_. (b) Double-reciprocal plots for determining the kinetic constants for the H_2_O_2_ substrate. (c) Velocity of the reaction measured by CuN_4_-CNS toward various concentrations of TMB. (d) Double-reciprocal plots for determining the kinetic constants for the TMB substrate. (e) Velocity of the reaction measured by CuN_4_-CNS toward various concentrations of CuN_4_-CNS. (f) Relative activities of CuN_4_-CNS with different pH values.


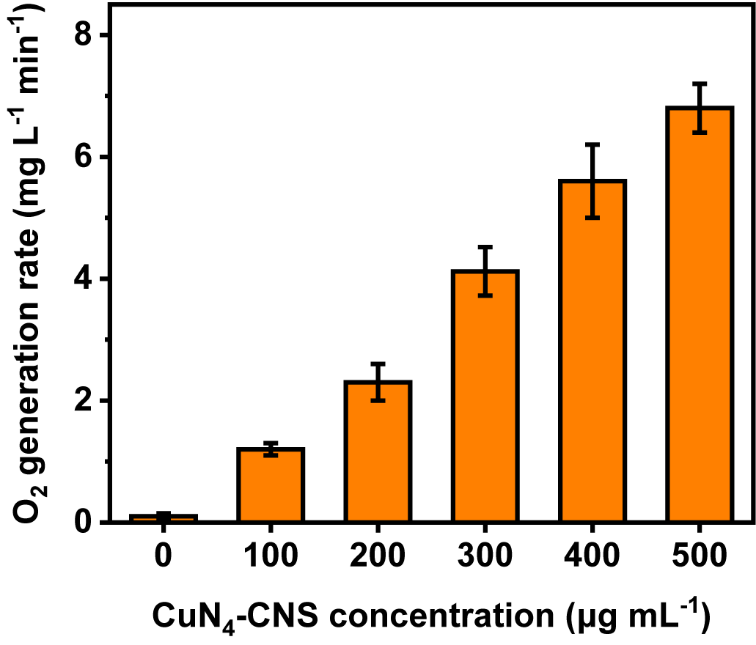


**Figure S10.** **O_2_ generation rates at different CuN_4_-CNS concentrations.**

**Figure S11. Fluorescence intensity of DHE treated with or without CuN_4_-CNS and CuN_2_-CNS in air-saturated solution.**


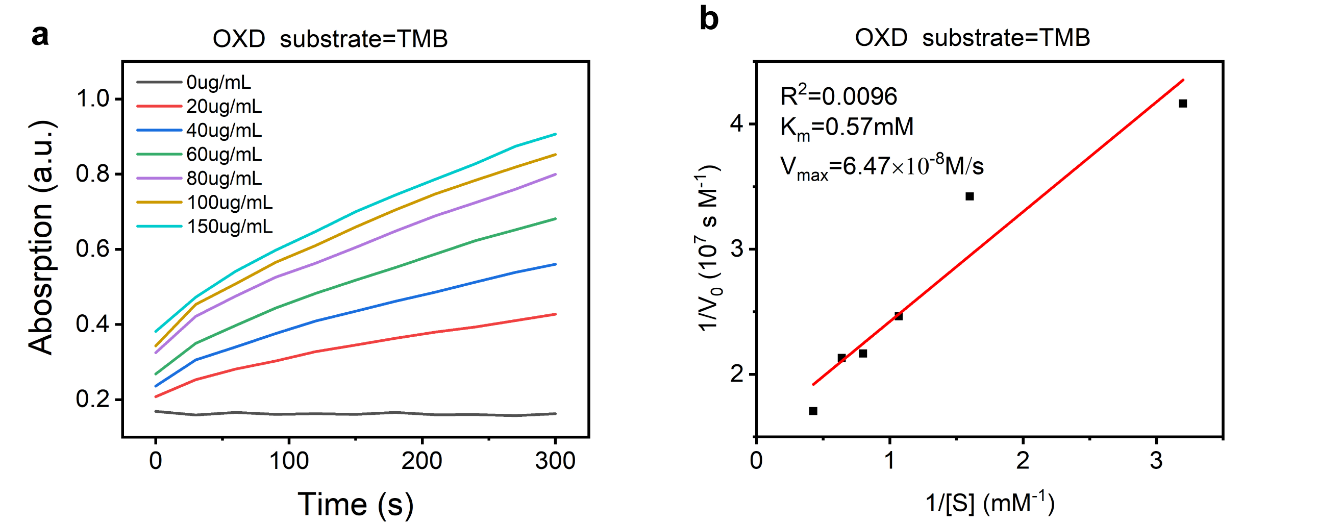


**Figure S12. Catalytic kinetics of CuN_4_-CNS with O2 as substrates.** (a) Velocity of the reaction measured by CuN_4_-CNS toward various concentrations of TMB. (b) Double-reciprocal plots for determining the kinetic constants for the TMB substrate.


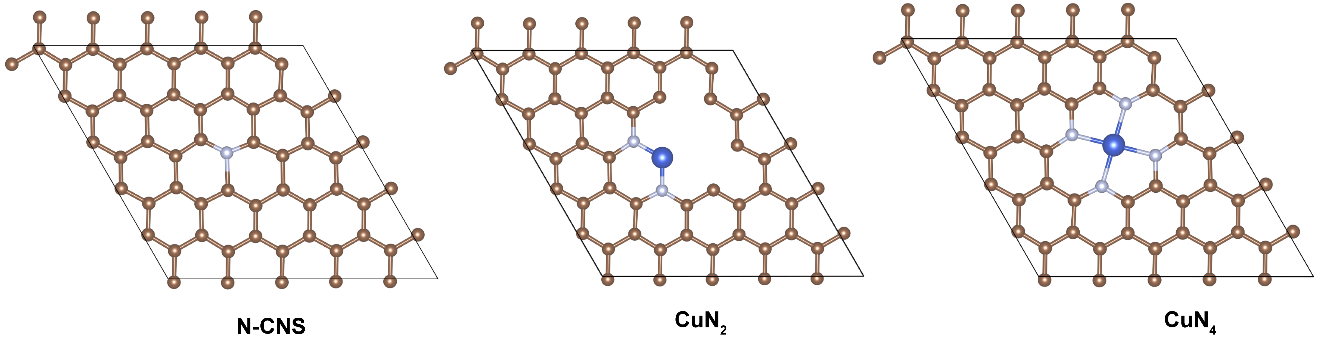


**Figure S13. Optimized structures of N-CNS, CuN_2_-CNS, CuN_4_-CNS based on XAFS analysis.**


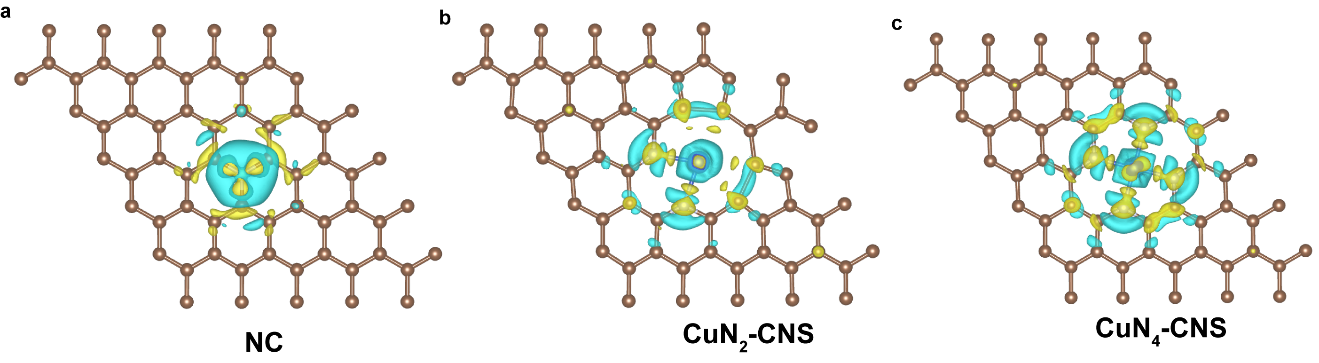


**Figure S14. Bonding charge distributions of the NC, CuN_2_ and CuN_4_ sites.**


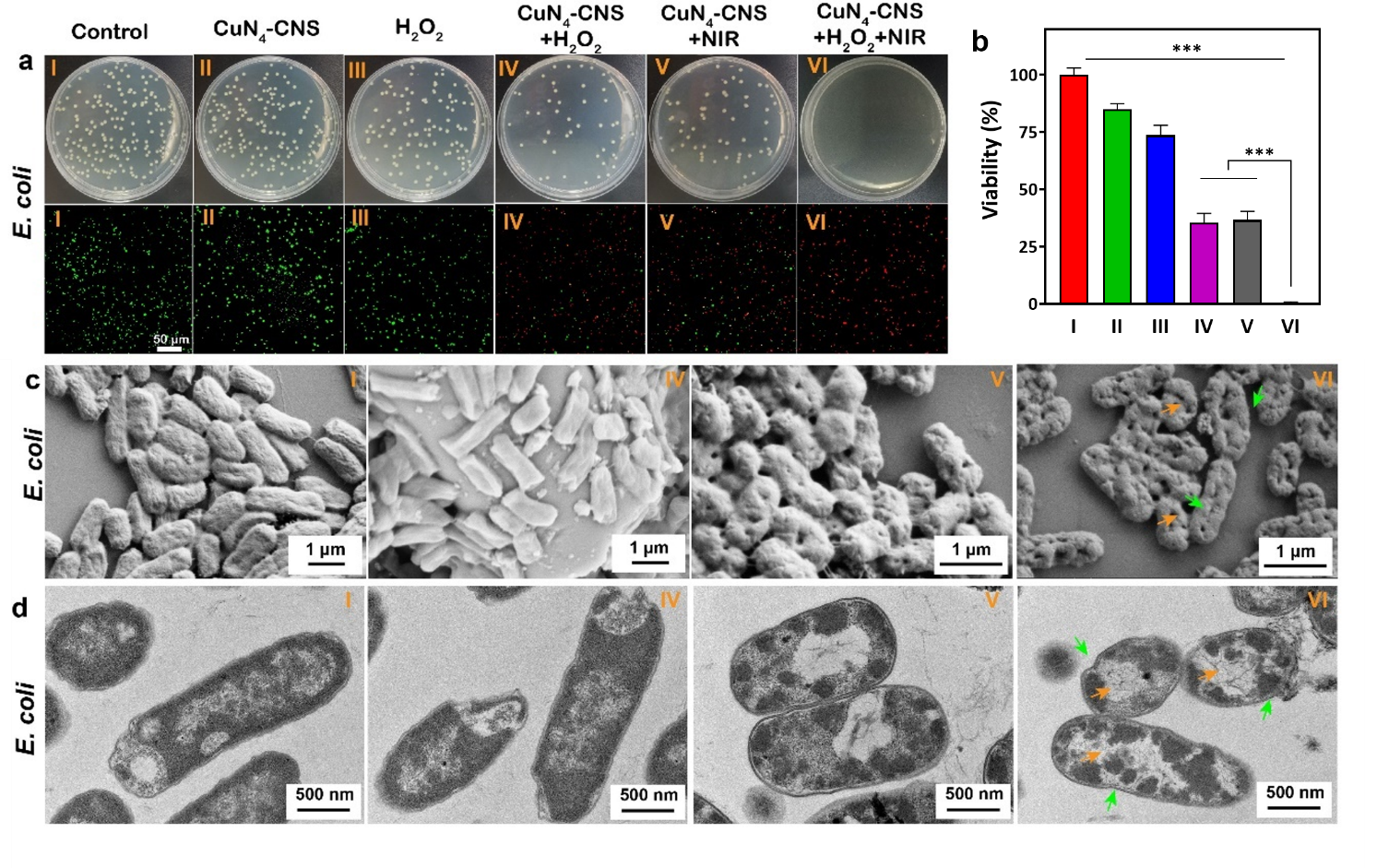


**Figure S15.** **Catalytic antibacterial performance of the CuN_4_-CNS for *E. coli*.** (a) Photographs of bacterial colonies formed by *E. coli* and SYTOX/PI staining results after treatment with PBS (I), CuN_4_-CNS (II), H_2_O_2_ (III), CuN_4_-CNS+H_2_O_2_ (IV), CuN_4_-CNS+NIR (V), CuN_4_-CNS+H_2_O_2_+NIR (VI) for 10 min. (b) Corresponding bacterial viability of E. coli. (n = 6; ***P < 0.001).


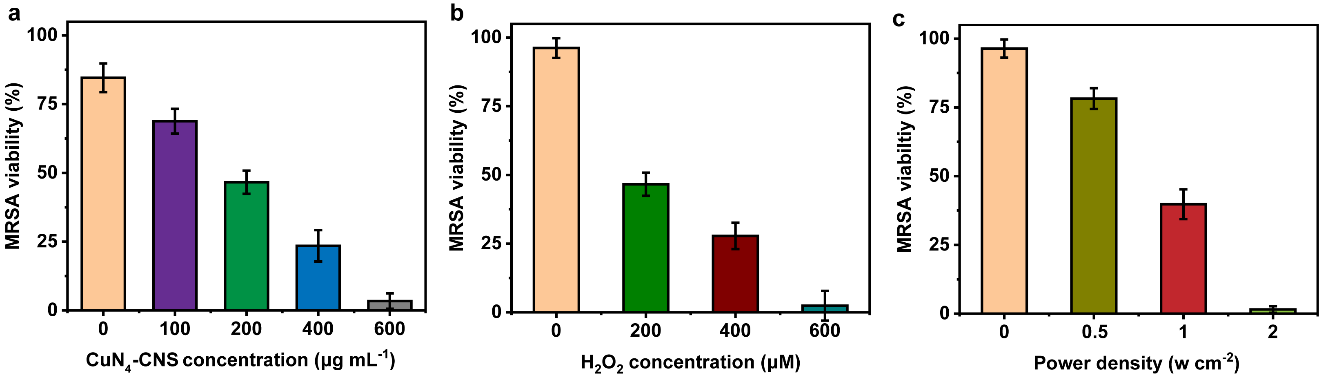


**Figure S16. MRSA viabilities** treated under different CuN_4_-CNS concentrations (a), H_2_O_2_ concentrations (b), and laser power densities (c). (n = 6; ***P < 0.001).


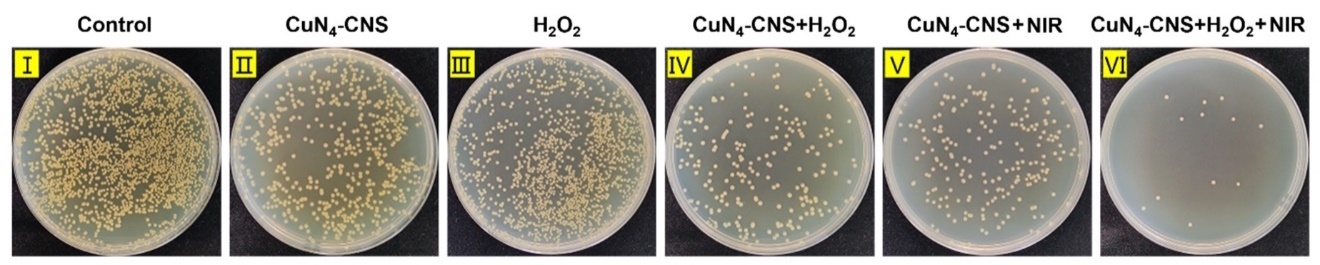


**Figure S17. Representative culture photographs of bacterial colonies from biofilms treated by different conditions.**


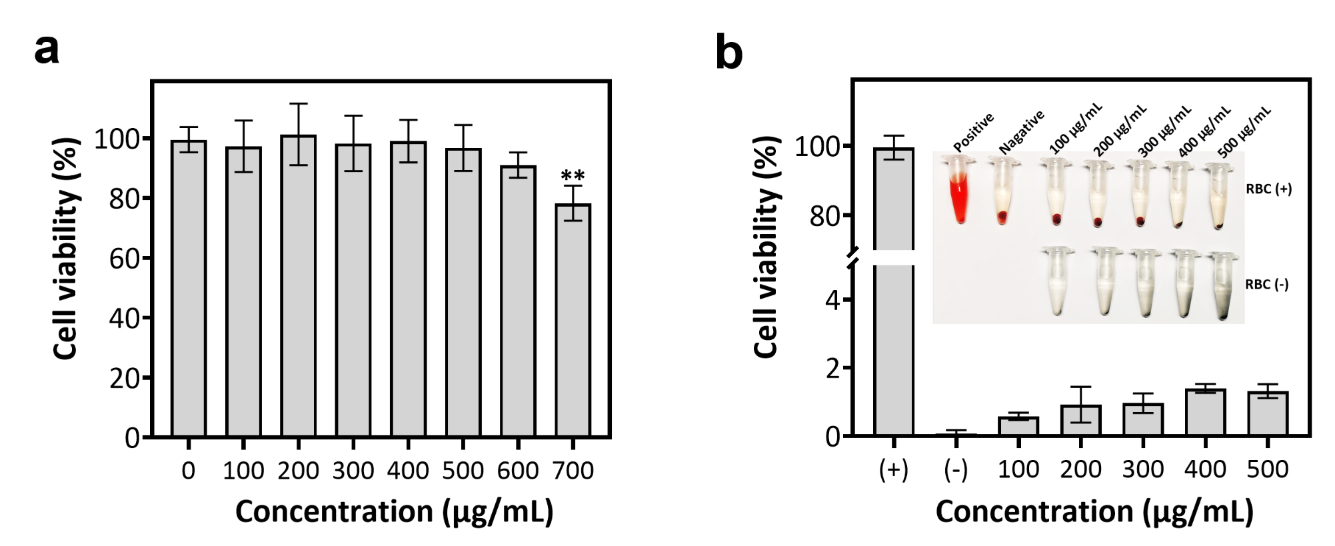


**Figure S18. In vitro biocompatibility of CuN_4_-CNS.** (a) Relative viability of HUVECs with different concentrations of CuN_4_-CNS. (b) Hemolysis test of CuN_4_-CNS. (n = 6; **P < 0.01).


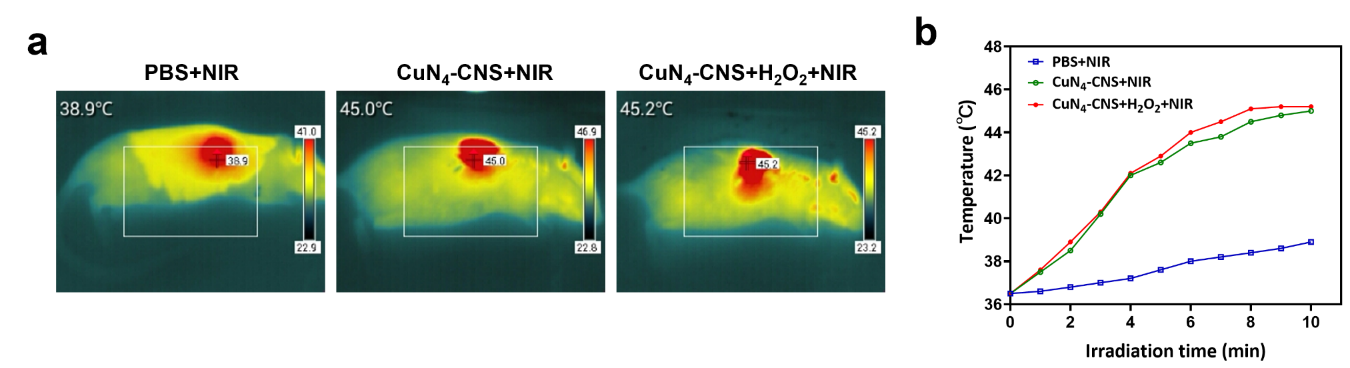


**Figure S19.** **Thermal images of rats.** (a) Thermal images of rats treated with CuN_4_-CNS before and after NIR laser irradiation (1064 nm, 1.0 W cm^2^, 10 min). (b) Temperature change curves of rats treated with CuN_4_-CNS after 1064 nm laser irradiation (1.0 W/cm^2^).

**Table S1.** **Specific surface areas (S_BET_) calculated by the BET method based on N_2_ adsorption and desorption measurements and elemental compositions of the CuN_x_-CNS estimated from XPS and ICP measurements.^a^**

| **Sample** | **S_BET_**  **(m^2^ g^-1^)** | **C (wt%)** | **O (wt%)** | **N (wt%)** | **Cu (wt%)** |
| --- | --- | --- | --- | --- | --- |
| N-CNS | 582 | 78.4 | 16.9 | 4.7 | 0 |
| CuN_4_CNS | 838 | 75.5 | 13.5 | 6.2 | 1.7 |
| CuN_2_-CNS | 796 | 78.2 | 15.2 | 5.1 | 1.5 |

^a^C, O, and N contents were detected by XPS, and Cu contents were determined by ICP.

**Table S2. EXAFS fitting parameters at the Cu K-edge for various samples (*Ѕ*_0_^2^= 0.84)**

| **Samples** | **shell** | **CN** | **R(Å)** | **σ^2^** | **ΔE_0_** |  | **R factor** |
| --- | --- | --- | --- | --- | --- | --- | --- |
| Cu foil | Cu-Cu | 12 | 2.54±0.01 | 0.0086 | 4.4±0.5 |  | 0.0026 |
| CuN_4_-CNS | Cu-N | 3.9±0.2 | 2.04±0.02 | 0.0151 | 5.7±2.1 |  | 0.0042 |
| CuN_2_-CNS | Cu-N | 2.4±0.1 | 1.94±0.01 | 0.0095 | 4.1±1.6 |  | 0.0099 |

*^a^N*: coordination numbers; *^b^R*: bond distance; *^c^σ*^2^: Debye-Waller factors; *^d^* Δ*E*_0_: inner potential correction. *R* factor: goodness of fit.

**Table S3. Comparisons of the activities of different SAzymes.**

| Catalysts | Substrates | K_M_ (mM) | V_max_  (10^-8^ M s^-1^) | References |
| --- | --- | --- | --- | --- |
| Fe_3_O_4_ nanozymes | TMB | 0.098 | 3.44 | [35] |
|  | H_2_O_2_ | 154 | 9.78 |  |
| HRP | TMB | 0.43 | 10.00 | [35] |
|  | H_2_O_2_ | 3.70 | 8.71 |  |
| 2D Fe-N-C SAzymes | TMB | 4.31 | 62.0 | [29] |
|  | H_2_O_2_ | 5.20 | 149.0 |  |
| Fe-N-C SAzymes | TMB | - | - | [36] |
|  | H_2_O_2_ | 0.012 | 22.3 |  |
| 2D Zn-N-C SAzymes | TMB | 6.27 | 0.48 | [29] |
|  | H_2_O_2_ | 0.28 | 0.43 |  |
| Zn-N-C SAzymes | TMB | 0.224 | 10.66 | [37] |
|  | H_2_O_2_ | 40.16 | 12.15 |  |
| 2D Cu-N-C SAzymes | TMB | 3.76 | 75.05 | [27] |
|  | H_2_O_2_ | 19.94 | 20.07 |  |
| Cu-N-C SAzymes | TMB | 0.07 | 1.25 | [28] |
|  | H_2_O_2_ | 0.25 | 1.5 |  |
| 2D CuN_4_-CNS SAzymes | TMB | 0.223 | 9.49 | This work |
|  | H_2_O_2_ | 5.63 | 138.0 |  |
